# Functionalization of Biphenylcarbazole (CBP) with Siloxane-Hybrid Chains for Solvent-Free Liquid Materials

**DOI:** 10.3390/molecules27010089

**Published:** 2021-12-24

**Authors:** Janah Shaya, Gabriel Correia, Benoît Heinrich, Jean-Charles Ribierre, Kyriaki Polychronopoulou, Loïc Mager, Stéphane Méry

**Affiliations:** 1 IPCMS, CNRS-Strasbourg University, UMR7504, 23 Rue du Loess, BP 43, 67034 Strasbourg, France; gabriel.correia@thermofisher.com (G.C.); benoit.heinrich@ipcms.unistra.fr (B.H.); loic.mager@ipcms.unistra.fr (L.M.); 2 Department of Chemistry, College of Arts and Sciences, Khalifa University, Abu Dhabi P.O. Box 127788, United Arab Emirates; 3 College of Medicine and Health Sciences, Khalifa University, Abu Dhabi P.O. Box 127788, United Arab Emirates; 4 Center for Organic Photonics and Electronics Research (OPERA), Kyushu University, 744 Motooka, Nishi, Fukuoka 819-0395, Japan; ribierre@opera.kyushu-u.ac.jp; 5 Department of Mechanical Engineering, Khalifa University of Science and Technology, Abu Dhabi P.O. Box 127788, United Arab Emirates; kyriaki.polychrono@ku.ac.ae; 6 Center for Catalysis and Separation, Khalifa University of Science and Technology, Abu Dhabi P.O. Box 127788, United Arab Emirates

**Keywords:** molecular liquid, allyl isomerization, Stille coupling, Ullmann coupling, hydrosilylation, liquid optoelectronics, liquid semiconductor

## Abstract

We report herein the synthesis of siloxane-functionalized CBP molecules (4,4′-bis(carbazole)-1,1′-biphenyl) for liquid optoelectronic applications. The room-temperature liquid state is obtained through a convenient functionalization of the molecules with heptamethyltrisiloxane chains via hydrosilylation of alkenyl spacers. The synthesis comprises screening of metal-catalyzed methodologies to introduce alkenyl linkers into carbazoles (Stille and Suzuki Miyaura cross-couplings), incorporate the alkenylcarbazoles to dihalobiphenyls (Ullmann coupling), and finally introduce the siloxane chains. The used conditions allowed the synthesis of the target compounds, despite the high reactivity of the alkenyl moieties bound to π-conjugated systems toward undesired side reactions such as polymerization, isomerization, and hydrogenation. The features of these solvent-free liquid CBP derivatives make them potentially interesting for fluidic optoelectronic applications.

## 1. Introduction

Siloxane-containing organic materials are widely used and have currently become essential for many sectors of activities such as cosmetics, paints, coatings, packaging as well as automotive, household and medical appliances. The siloxane moiety enables to confer unique surface, mechanical, structural and thermal properties [1,2,3].

Recently, there is a growing interest for siloxane-functionalized materials for optoelectronic applications [4,5,6,7,8,9,10,11,12,13,14]. This trend might be surprising because of the electrical insulating character of siloxanes. However, the incorporation of short siloxane segments into electro-active conjugated species enables a valuable improvement of the organization, thermal and mechanical properties of the materials. For instance, siloxane-hybrid side-chain conjugated polymers have been reported to exhibit lamellar mesomorphic organization with enhanced charge transport properties [11,12,13,14]. This effect was attributed to the singular fluid and segregating character of siloxane chains that impose a better facing of the polymers with improved π-stacking overlap [14]. Multiple functionalization of conjugated molecular systems by short siloxane chains also led to preparation of molecular liquids with luminescent and/or semiconducting properties [4,5,6,7,8,9]. Here, the tight connection of flexible and bulky siloxane segments allows a significant reduction of the π-stacking interactions to prevent crystallization and confer a stable liquid state at room temperature. Thus, siloxane-chain functionalization appears as an excellent alternative to classical branched alkyl chain functionalization for designing solvent-free functional molecular liquids (FMLs) [4,15,16]. FMLs are quite promising materials for optoelectronics and the first proof of concepts have already been reported in fluidic OLEDs [17,18], in dye-sensitized solar cells [7], and in optical lasers [9], in particular. As compared to molecules in solution, FMLs offer the unique advantages of being stable homogeneous liquids that are nonvolatile and devoid of aggregates [15,16]

For chemical stability reasons, the siloxane segment is usually not directly connected to the π-conjugated moiety, but through an alkyl linker. The use of a short alkyl linker is advantageous for a proximal connection of the sterically demanding siloxane chains to design molecular liquids, in particular [9]. The functionalization is commonly performed by the hydrosilylation of a silane (Si-H) functionality of the siloxane chain to a ω-alkene [19,20]. When alkyl linkers as short as vinylene or propylene are used, the functionalization becomes rather untrivial because the vinyl- and allyl-aryl intermediates turn to be highly sensitive to side-reactions (i.e., polymerization, hydrogenation or isomerization) [20,21,22]. Therefore, developing synthetic strategies to obtain vinyl- and allyl-aryl systems and their subsequent siloxane-functionalized derivatives is still needed.

**Figure 1:**
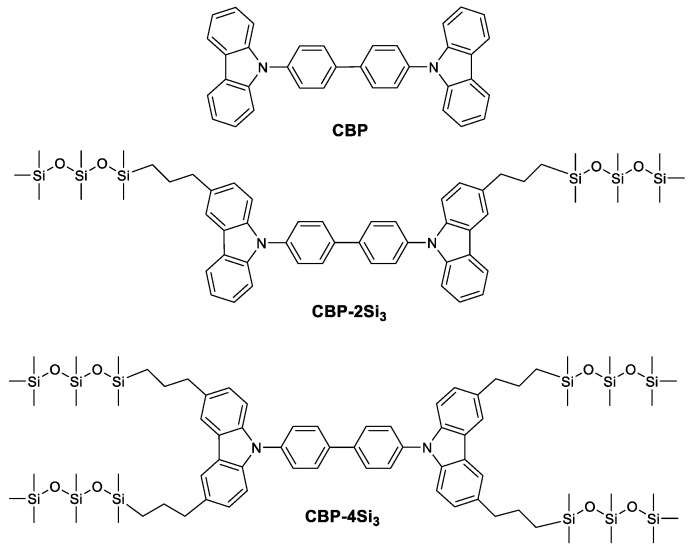
Molecular structures of CBP and siloxane-functionalized CBP derivatives.

To study these fundamental aspects, we focused on the siloxane functionalization of a carbazole derivative extensively used in optoelectronics, namely 4,4′-bis(carbazole)-1,1′-biphenyl (CBP). CBP belongs to the broad family of carbazole-based materials developed for OLEDs, photovoltaic devices, gas storage and separation, among other applications [23,24,25,26,27,28]. CBP is most known as semiconducting host in OLED applications [29]. Its favorable high triplet state and good bipolar charge transport characteristics in its metastable glassy state already led to light emitting diodes of high quantum efficiencies [30,31]. Despite these good results, CBP still suffers from poor film stability due to its strong crystallization tendency with time [32]. The important uses of CBP and its stability issue inspired us to develop synthetic approaches to obtain challenging siloxane-functionalized CBP materials of liquid character for potential uses in fluidic optoelectronic devices (Figure 1).

The great advances in catalytic reactions such as photocatalysis, homogeneous catalysis, and heterogeneous catalysis are now allowing synthesis of otherwise challenging materials for all applications from environmental treatment to medicinal and materials sciences [33,34,35,36,37,38,39]. From a synthetic point of view, the introduction of a siloxane chain to a compound of interest usually requires starting the synthesis from the beginning. Following modern catalytic routes, CBP molecules are commonly synthesized using Buchwald−Hartwig or Ullmann-type coupling reactions between dihalobiphenyl molecules and a naked carbazole molecule [40,41,42,43]. However, the addition of alkenyl linkers for introducing siloxane chains to these conjugated cores, particularly those activated with heteroatoms such as carbazoles brings elevated complexity due to unfavored side reactions [21,22]. Among them, metal-catalyzed polymerization and isomerization were well documented in such aromatic systems in the literature [44,45,46,47,48]. Herein, we present a synthetic methodology to obtain CBP molecules functionalized by siloxane chains using a short ω-alkenyl (essentially allyl) linker. The main synthetic route comprised incorporating the alkenyl linker to bromocarbazoles via Pd-catalyzed reactions such as Stille and Suzuki-Miyaura cross-coupling reactions [21,33,49,50]. The alkenylcarbazoles were introduced to dihalobiphenyls using Ullmann coupling. Lastly, the hydrosilylation of the alkenyl group with 1,1,1,3,3,5,5-heptamethyltrisiloxane was planned using Karstedt’s platinum catalyst [51,52].

## 2. Results and Discussion

**Scheme 1:**
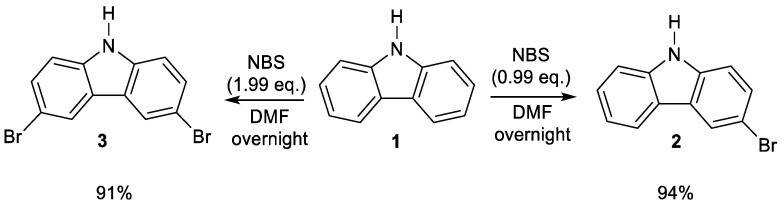
Bromination of carbazole.

The synthesis of the functionalized carbazole-based derivatives with siloxane chains (shown in Figure 1) started by previously reported radical halogenation of carbazole **1** with *N*-bromosuccinamide in DMF (Scheme 1) [53,54]. 3-bromocarbazole **2** and 3,6-dibromocarbazole **3** products were easily obtained by controlling stoichiometrically the amount of NBS (0.99 eq. for **2** and 1.99 eq. for **3**). Simple recrystallization in ethanol afforded the pure products in 91% and 94%, respectively.

Then, we envisaged incorporating the allyl linker using Stille coupling, given the known versatility and stability of tin reagents [55,56]. Catalytic conditions on 3-bromocarbazole **2** were used to dodge side Pd-catalyzed reactions such as homocoupling, reductive dehalogenation, Buchwald–Hartwig amination, and isomerization of the allyl substituent [21,22,44,45,46,47,48]. Trials were performed on a small scale (50.0 mg, 0.203 mmol) and characterized by GC/MS conversions as shown in Table 1.

Pd(PPh_3_)_4_ did not result in the selective mono-allyl coupling on the C3 position, rather it produced a mixture of isomers of N,3-bisallylcarbazole **6** (entry 1). In contrast, PdCl_2_ with PPh_3_ ligand avoided the allylation on the nitrogen. Pd(PPh_3_)_4_ in situ formation prior to allyl coupling could have slowed down the reaction kinetics which limited the formation of poly-coupling side products [57]. The optimal conditions and catalytic loading were found to be as follows: PdCl_2_ (3.5 mol%), PPh_3_ (14.0 mol%), tributylallylstannane (1.2 eq.) in DMF at 100 °C (entry 3). This one-step coupling represents an advantage versus the expected three-step route, in which protection/deprotection of the N-H group was needed to avoid its allylation by stannyl reagents [58]. The optimized conditions afforded complete conversion with around 1% of the isomerized allylcarbazole **5**. Higher catalytic loadings (6.0 mol%) favored the competitive isomerization leading to product **5** (8%, entry 4). Lower Pd content (2.5 mol%) led to incomplete conversion with relatively high percentage of the reductive dehalogenation product **1** (4%) and without eliminating isomer **5** (entry 2). Longer reaction time (2–3 days) did not produce better results. In addition, using non-polar, non-coordinating solvents such as toluene was less efficient in this catalytic cycle and afforded 77% conversion to the desired product (entry 5).

**Scheme 2:**
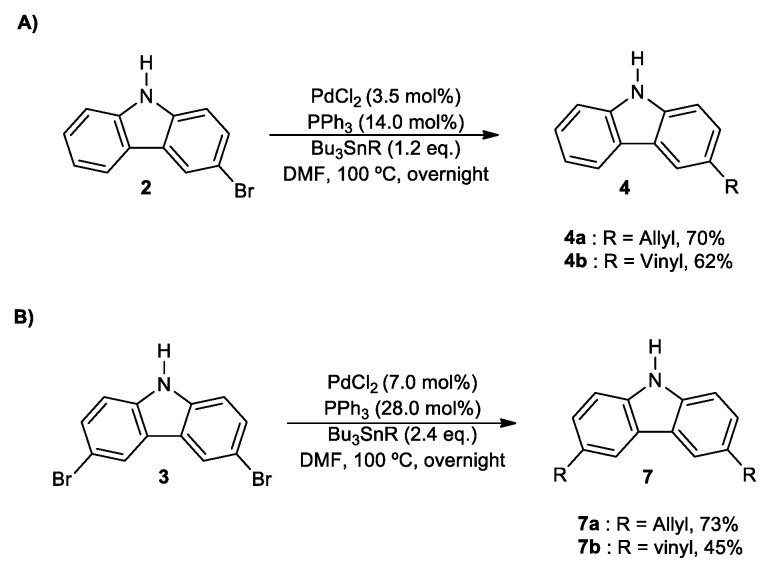
Synthesis of mono substituted (**A**) and di-substituted (**B**) carbazole derivatives bearing vinyl or allyl pendant.

Once we had the coupling conditions in hand, we shifted to the synthesis of the allylcarbazole intermediates (Scheme 2). Monoallyl and monovinyl carbazoles **4a**–**b** were obtained in good yields (70% and 62% respectively). 3,7-bisallylcarbazole **7a** was prepared with 73% yield by doubling the catalytic loading and the allyltributylstannane. The purification of this product **7a** was tedious as the higher amount of palladium in combination with the extended conjugation of the carbazole moiety with two allyl pendants triggered the formation of 5–10% of the isomerized allyl product. The two formed products have the same *R*_f_ in almost all the eluents, which make them difficult to separate. Besides, it was observed that the simple use of rotary evaporator at temperature greater than 30 °C might result in isomerization of the allyl groups on bisallylcarbazole. Isolation of the product with purity above 99% could only be achieved by repetitive column chromatography with silica/sample ratio greater than 400/1 while monitoring by GC/MS and not by thin layer chromatography. It seems that the extended conjugation on this aromatic moiety bearing a (polarizable) heteroatom (N-H) increases the electrostatic interactions among the molecules rendering the separation process highly complicated [46,48,59].

The 3,7-bisvinylcarbazole **7b** was easier to isolate in comparison to the allyl **7a** product due to the absence of possible isomers. The isolated yield was moderate (45%) since this intermediate is apt to polymerization. Some reports showed that the use of free radical inhibitors such as BHT can further increase the yield of such reaction [60].

**Scheme 3:**
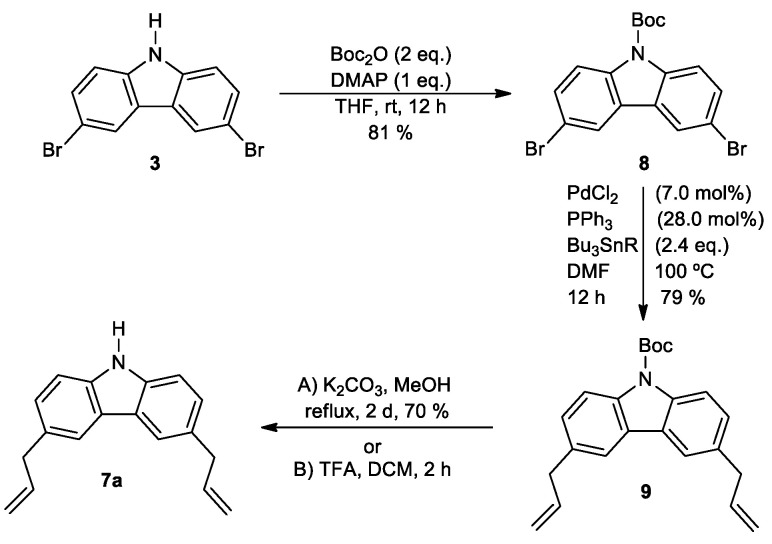
Synthesis of 3,6-bisallylcarbazole via Boc-protected intermediate.

Next, we conducted additional trials to probe if side reactions can be avoided, particularly isomerization. The bisallyl derivative **7a** was synthesized using *N*-Boc protected carbazole via three-step route (Scheme 3). Conventional Boc protection using Boc_2_O and DMAP in THF produced adduct **8** in 81% yield [61]. Stille coupling on **8** led to the bisallyl intermediate **9** with a good yield (79%). In the final Boc-deprotection step, two conditions were tested. Trifluoroacetic acid in DCM was not fruitful and led to a mixture of products. Milder deprotection conditions using K_2_CO_3_ in MeOH resulted in the desired product **3a** (70%). Nevertheless, 5–10% isomerization of the allyl linkers was still encountered, thus adding the same difficulty in separation as in the case of the one-step route, which makes the previous synthetic route more favorable.

**Scheme 4:**
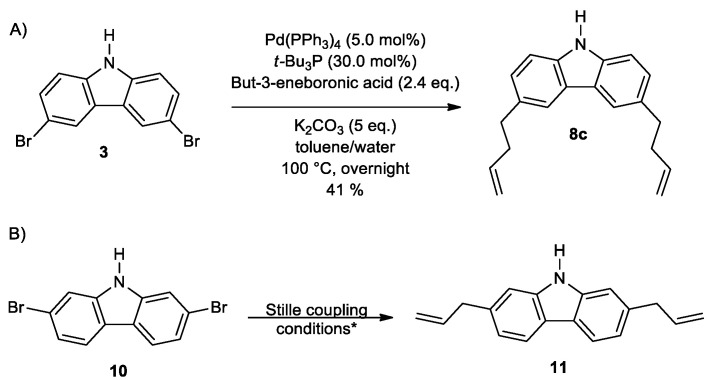
Final tests of isomerization of the butenyl and allyl pendants on para (**A**) and meta (**B**) positions of carbazole. * The Stille coupling conditions are the same as Scheme 3A.

To further investigate the isomerization of the alkenyl moiety, we tested Suzuki-Miyaura coupling of butenyl linker using conventional conditions for this type of reactants from the literature; compound **8c** (Scheme 4A). 3,6-bisbuten-1-ylcarbazole **8c** was obtained with a lower yield (41%) than those of Stille coupling. We then investigated the incorporation of the allyl group on 2,7-positions of the carbazole (meta-bisallylcarbazole **11** (Scheme 4B) using the optimized conditions of Stille coupling (as in Scheme 3). Similar GC/MS profile to that of the ortho-bisallylcarbazole previously discussed was obtained showing isomerized allyl side products. It can be concluded that the position of the allyl linker (meta or para) has little impact on its isomerization pathway during this catalytic cycle.

**Scheme 5:**
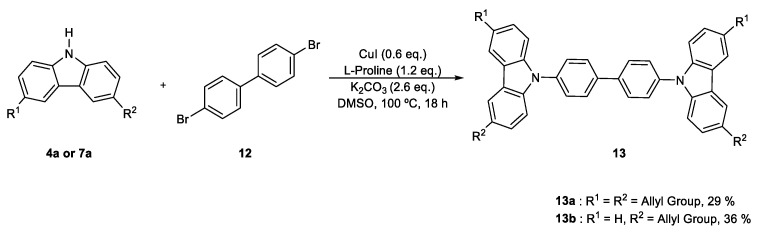
Synthesis of carbazolebiphenyl derivatives bearing the allyl pendants.

In the second step, we selected conditions for coupling unsubstituted carbazole to a dihalobiphenyl core from literature [28,41,43,62]. We performed small-scale trials on naked carbazoles and chose the most promising conditions by thin layer chromatography to introduce the allylcarbazoles. The isomerization of the allyl pendant was the major drawback as evidenced by crude ^1^H-NMR. It was observed that leaving the reaction to proceed to completion (3 days), or re-adding a second catalytic loading, or else heating above 100 °C all promoted the alkenyl isomerization resulted in a mixture of products impossible to separate. Different catalysts were examined (CuI, Cu, Pd(OAc)_2_), bases (K_2_CO_3_ or Cs_2_CO_3_), additives (proline, phenanthroline, LiCl, crown ether), solvents (DMF, DMSO, orthodichlorobenzene). Isomerization could not be circumvented using all these conditions. However, when the Ullmann coupling was carried out using CuI (0.6 eq.), l-Proline (1.2 eq.), potassium carbonate (2.6 eq.) and 3-allylcarbazole (2.3 eq.) in DMSO at 100 °C and stopped after 18 h, the desired product was formed and could be isolated in low yield without pushing the reaction to completion to avoid isomerization (Scheme 5).

The crude mixture showed a majority of mono-coupled carbazole and starting material that could be recovered. This compromise in yield permitted us to obtain the novel and versatile intermediates **13a** in 29% yield (20% mono-coupled carbazole and 40% recovered allylcarbazole) and **13b** in 36% yield (15% mono-coupled carbazole and 38% recovered starting material). No significant increase in yields was observed when diiodobiphenyl was used instead of dibromobiphenyl. Conversely, Ulmann coupling on the bisvinylcarbazole **4b** completely led to polymerization.

**Scheme 6:**
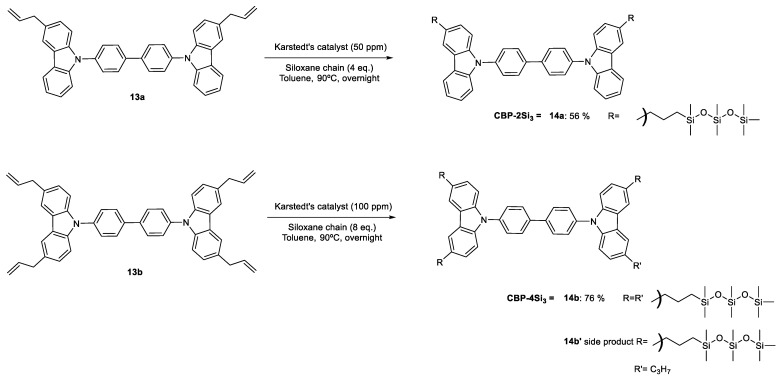
Hydrosilylation of the bis- and tetra-allyl-substituted CBP derivatives.

In the final step, hydrosilylation was carried out using Karstedt’s catalyst to transform the mono and bisallylcarbazole-biphenyl **13a**–**b** to the corresponding siloxane-functionalized CBP (**14a**–**b**) [51,52]. A low Karstedt’s catalytic loading (50 ppm/C=C) successfully led to the preparation of the disiloxane **14a** (**CBP-2Si_3_**) in 56% yield (Scheme 6). **CBP-2Si_3_** was a soft solid at room temperature inferring that two heptamethyltrisiloxane chains are not enough to change CBP into liquid state.

The hydrosilylation of the biphenyl bearing bisallylcarbazole **13b** was more challenging. When this reaction was carried out on a small scale on **13b** (50.0 mg, 0.078 mmol), the pure liquid product **14b** (**CBP-4Si_3_**) was isolated in 76% yields (Scheme 6). Performing the same reaction on a larger scale, product **14b** was always achieved in good yields (above 70%), but it showed less than 5% of the side product **14b’**. The latter results from a competitive Pt-catalyzed hydrogenation of one of the four allyl chains. Its proportion can be limited to low content by using a low Pt loading. Nevertheless, the presence of side product **14b’** at low content is not problematic for applications as it does not affect the room-temperature liquid state of compound **14b** (**CBP-4Si_3_**), nor should it significantly affect its charge transport and optoelectronic properties.

**Figure 2:**
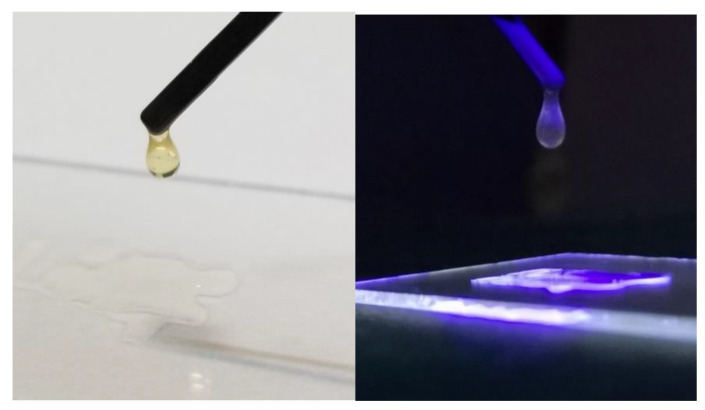
Photographs of **CBP-4Si_3_** under ambient visible light (**right**) and ultraviolet light (**left**).

**Figure 3:**
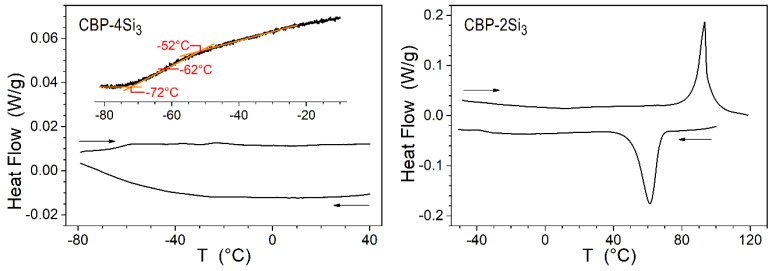
Differential scanning calorimetry (DSC) thermograms of the silylated compounds **14a** (**CBP4Si_3_**) and **14b** (**CBP-2Si_3_**) recorded on heating and cooling at 2 °C/min for **14a** and at 5 °C/min for **14b** (endotherm up); inset view: modulated DSC thermogram of the glass transition range of **14a** (modulation period: 40 s; amplitude: ±0.106 °C; ramp: 0.1 °C/min; ordinate: reversing heat flow, endotherm up). **CBP-4Si_3_** is a liquid with the glass transition located at −62 °C whereas **CBP-2Si_3_** is a solid melting at 87 °C and crystallizing again from liquid on cooling.

The siloxane chain functionalization of the biphenyl biscarbazole derivative could successfully confer the molecules with a liquid state at room temperature. While the nude 4,4′-Bis(carbazole)-1,1′-biphenyl conjugated compound (CBP) is crystalline and exhibits a high melting point at about 285 °C, the incorporation via a propylene spacer of four heptamethyltrisiloxane chains (in **14b**, i.e., **CBP-4Si_3_**), were enough to lead to liquid character at room temperature. The reduction of the number of the heptamethyltrisiloxane chains down to two (in **14a**, i.e., **CBP-2Si_3_**) was not sufficient to induce a room temperature liquid state, but nevertheless lowered drastically the melting point to 87 °C. The liquid state in **14b** (**CBP-4Si_3_**) was confirmed by visual observation (Figure 2). It was also found to be indefinitely stable at room temperature and no crystallization was observed in sub-ambient conditions by differential scanning calorimetry (DSC) analysis (Figure 3). Therefore, the thermograms of compound **CBP-4Si_3_** clearly show the absence of any thermal event, except for the glass transition temperature (Tg) at low temperature, i.e., at about −60 °C.

## 3. Conclusions

To sum up, two siloxane-functionalized carbazole derivatives were synthesized. The carbazole derivative was CBP molecule and it was functionalized with 2 and 4 heptamethyltrisiloxane chains. Different short alkylene linkers (i.e., ethylene, propylene and butylene) were tested to connect closely the siloxane moiety to the carbazole moieties. The side-reactions inherent to the short alkylene spacer (i.e., polymerization, hydrogenation or isomerization of the ω-alkenyl intermediate) could successfully be circumvented through the optimization of a selected sequence of efficient transition metal-catalyzed reactions: (i) a Stille cross-coupling reaction to insert the ω-alkylene chain onto the carbazole unit, (ii) Ullmann coupling to build the CBP core multifunctionalized by ω-alkylene chains, and (iii) hydrosilylation to incorporate the terminal siloxane chains. The careful optimization of these reactions, in particular through the nature and the molar ratio of the transition metal catalyst, allowed significant reduction of the side reactions to obtain the targeted materials. Finally, the presence of the siloxane chains drastically affected the organization of the CBP molecules. As an example, the CBP core tetra-substituted by heptamethylsiloxane chains (via a propenyl linker) showed a flowing liquid state at room temperature. This result demonstrates the powerful action of the siloxane chain functionalization to control the π-stacking interactions, up to confer large π-conjugated molecular systems with a stable liquid state.

## 4. Materials and Methods

All reagents and chemicals were purchased from commercial sources (Aldrich, Across, Fluka) and used without further purification. Karstedt’s catalyst represents platinumdivinyl tetramethyldisiloxane complex with 2.0–2.5% Pt content in xylene. All reactions involving water- or air-sensitive material were performed in oven-dried glassware under argon by using Schlenk techniques employing double-line argon-vacuum lines and dry solvents. The reactions were monitored simultaneously by gas chromatography (GC/MS) and by thin-layer chromatography and visualized both by UV radiation (254 and 365 nm) and by spraying with relevant staining agent (KMnO_4_). ^1^H and ^13^C NMR spectra were recorded on a Bruker Avance 300 and a Bruker 400 Ultrashield^TM^ NMR spectrometers, with an internal lock on the 2H-signal of the solvent. Chemical shifts (δ) are given in ppm to the nearest 0.01 (^1^H) or 0.1 ppm (^13^C{^1^H}NMR) (recorded with complete proton decoupling and written as ^13^C in the experimental part for simplicity). The coupling constants (*J*) are given in Hertz (Hz). The signals are reported as follows: (s = singlet, d = doublet, t = triplet, quint = quintet, m = multiplet). Mass spectra analyses were performed by using a Maldi-TOF or GC/MS. The former was carried out on a time-of-flight mass spectrometer (MALDI-TOF-TOF Autoflex II TOF-TOF, Bruker Daltonics, Bremen, Germany) equipped with a nitrogen laser (λ = 337 nm). The latter was carried out on a gas chromatograph coupled with an electron ionization mass spectrometer 7090-5975C from Agilent Technologies. Differential Scanning Calorimetry (DSC) measurements were performed with a Q1000 equipment from TA Instruments, operated in standard and modulated modes.

*3-Bromocarbazole* (**2**), To a stirred solution of carbazole (100.0 mg, 0.574 mmol, 1 eq.) in DMF (1.0 mL) *N*-bromosuccinimide (102.2 mg, 0.568 mmol, 0.99 eq.) dissolved in DMF (1.0 mL) was added dropwise at 0 °C. The mixture was allowed to warm to room temperature and stirred overnight. White precipitates were formed after the mixture was poured into 5.0 mL of water. The mixture was allowed to warm to room temperature and was stirred overnight. White precipitates were formed after the mixture was poured into water. Precipitates were filtered and dissolved in dichloromethane. The organic layer was washed with water to remove water-soluble impurities. The organic fraction was dried over anhydrous magnesium sulphate and the solvent was removed under reduced pressure. The resulting white solid was purified by recrystallization from ethanol to give **2** as white crystals (132.8 mg, 0.540 mmol, 94%). *R*_f_ = 0.11 (Cy/DCM = 8:2); ^1^H NMR (300 MHz, DMSO, δ): 7.17 (t, ^3^*J* = 7.5 Hz, 1H, CH), 7.39–7.51 (m, 4H, CH), 8.16 (d, ^3^*J* = 7.5 Hz, 1H, CH), 8.35 (s, 1H, CH), 11.42 (s, 1H, NH); GC-MS (*m*/*z*): 245.0 [M]^+^.

*3,6-Dibromocarbazole* (**3**), Same procedure as 3-bromocarbazole (**2**), starting from carbazole (100.0 mg, 0.574 mmol, 1 eq.) and *N*-bromosuccinimide (205.4 mg, 1.143 mmol, 1.99 eq.) providing **3** (169.8 mg, 0.522 mmol, 91%). *R*_f_ = 0.27 (Cy/DCM 6:4); ^1^H NMR (300 MHz, DMSO, δ): 7.46–7.55 (m, 4H, CH), 8.43 (s, 2H, CH), 11.60 (s, 1H, NH); GC-MS (*m*/*z*): 324.9 [M]^+·^.

*3-Allylcarbazole* (**4a**), 3-bromocarbazole (100.0 mg, 0.402 mmol, 1 eq.), PdCl_2_ (2.5 mg, 3.5 mol%) and PPh_3_ (14.9 mg, 14.0 mol%) were added to a previously dried reaction tube containing a magnetic bar. The tube was purged with argon for three cycles using Schlenk technique. The mixture was dissolved in 1.3 mL of DMF and stirred for 15 min under argon at rt. Then, allyltributylstannane (154 μL, 0.483 mmol, 1.2 eq.) was added, followed by overnight stirring at 100 °C. The reaction was concomitantly monitored by TLC and GC/MS until complete conversion. Finally, the reaction was cooled down to room temperature, quenched with NaOH (1N), and stirred for 5 min. The organic layer was extracted with DCM (2x) and dried over magnesium sulphate. The volatiles were removed under reduced pressure, and the residue was purified by silica gel column chromatography with Cy/DCM (8:2 *v*/*v*) as eluent providing the desired product **4a** (58.0 mg, 0.280 mmol, 70%). *R*_f_ = 0.33 (Cy/DCM 6:4); ^1^H NMR (300 MHz, CDCl_3_, δ): 3.49 (d, ^3^*J* = 6.5 Hz, 2H, CH_2_), 5.00–5.09 (m, 2H, CH_2_), 6.01 (ddt, ^3^*J*_trans_ = 16.5 Hz, ^3^*J*_cis_ = 10.0 Hz, ^3^*J* = 6.5 Hz), 7.11–7.32 (m, 5H, CH), 7.81 (s, 1H, CH), 7.84 (br s, 1H, NH), 7.97 (d, ^3^*J* = 8.0 Hz, 1H, CH); ^13^C NMR (75 MHz, CDCl_3_, δ): 40.7 110.8, 110.9, 115.7, 119.6, 120.3, 120.6, 123.6, 123.9, 126.1, 127.1, 131.4, 138.5, 138.8, 140.6; GC-MS (*m*/*z*): 207.1 [M]^+^.

*3-Vinylcarbazole* (**4b**), Same procedure as 3-allylcarbazole (**4a**), starting from 3-bromocarbazole (100.0 mg, 0.402 mmol, 1 eq.), PdCl_2_ (2.5 mg, 3.5 mol%) and PPh_3_ (14.9 mg, 14.0 mol%) and vinyltributylstannane (145 μL, 0.483 mmol, 1.2 eq.), providing **4b** (48.3 mg, 250 mmol, 62%). *R*_f_ = 0.33 (Cy/DCM 6:4). ^1^H NMR (300 MHz, CDCl_3_, δ): 5.22 (d, ^3^*J*_cis_ = 11.0 Hz, 1H, CH), 5.79 (d, ^3^*J*_trans_ = 17.5 Hz, 1H, CH), 6.92 (dd, ^3^*J*_trans_ = 17.5 Hz, ^3^*J*_cis_ = 11.0 Hz, 1H, CH), 7.23–7.28 (m, 1H, CH), 7.36 (d, ^3^*J* = 8.5 Hz, 1H, CH), 7.41–7.43 (m, 2H, CH), 7.55 (dd, ^3^*J* = 8.5 Hz, ^4^*J* = 1.5 Hz, 1H, CH), 8.02 (br s, 1H, NH), 8.08 (s, 1H, CH), 8.11 (s, 1H, CH); GC-MS (*m*/*z*): 193.1 [M]^+^.

*3,6-Bisallylcarbazole* (**7a**), Same procedure as 3-allylcarbazole (**4a**), starting from 3,6-dibromocarbazole (100.0 mg, 0.305 mmol, 1 eq.), PdCl_2_ (3.8 mg, 7.0 mol%), PPh_3_ (22.6 mg, 28.0 mol%) and allyltributylstannane (234 μL, 0.731 mmol, 2.4 eq.) providing **7a** (55.0 mg, 0.199 mmol, 73%). *R*_f_ = 0.33 (Cy/DCM 6:4); ^1^H NMR (300 MHz, CDCl_3_, δ): 3.48 (d, ^3^*J* = 6.5 Hz, 4H, CH_2_), 5.02 (m, 4H, CH_2_), 6.00 (ddt, ^3^*J*_trans_ = 16.5 Hz, ^3^*J*_cis_ = 10.0 Hz, ^3^*J* = 6.5 Hz, 2H, CH), 7.14–7.27 (m, 4H, CH), 7.79 (s, 2H, CH), 7.81 (br s, 1H, NH); ^13^C NMR (75 MHz, CDCl_3_, δ): 40.7, 110.8, 115.6, 120.3, 123.8, 127.0, 131.3, 138.8, 138.8; GC-MS (*m*/*z*): 247.2 [M]^+·^. MS (Maldi-TOF) (*m*/*z*): 247.161.

*3,6-Bisvinylcarbazole* (**7b**), Same procedure as 3-allylcarbazole (**4a**), starting from 3,6-dibromocarbazole (100.0 mg, 0.305 mmol, 1 eq.), PdCl_2_ (3.8 mg, 7.0 mol%), PPh_3_ (22.6 mg, 28.0 mol%) and vinylltributylstannane (213 μL, 0.732 mmol, 2.4 eq.), providing **7b** (30.0 mg, 0.138 mmol, 45%)**.** *R*_f_ = 0.33 (Cy/DCM 6:4). ^1^H NMR (300 MHz, CDCl_3_, δ): 5.21 (d, ^3^*J*_cis_ = 11.0 Hz, 2H, CH), 5.78 (d, ^3^*J*_trans_ = 17.5 Hz, 2H, CH), 6.90 (dd, ^3^*J*_trans_ = 17.5 Hz, ^3^*J*_cis_ = 11.0 Hz, 2H, CH), 7.35 (d, ^3^*J* = 8.5 Hz, 2H, CH), 7.52 (d, ^3^*J* = 8.5 Hz, 2H, CH), 8.09 (s, 2H, CH), 8.15 (br s, 1H, NH); ^13^C NMR (75 MHz, CDCl_3_, δ): 110.9 (2C), 111.5 (2C), 118.5 (2C), 123.7 (2C), 124.5 (2C), 129.8 (2C), 137.6 (2C), 139.9 (2C); GC-MS (*m*/*z*): 219.1 [M]^+^.

*N-Boc-3,6-dibromocarbazole (***8**), 3,6-dibromocarbazole (100.0 mg, 0.302 mmol, 1 eq.), di-tert-butylcarbonate (133.0 mg, 0.603 mmol, 2 eq.) and 4-(dimethylamino)pyridine (37.2 mg, 0.302 mmol, 1 eq.) were added to a flask containing a magnetic bar. The mixture was dissolved in 5 mL of THF and stirred for 12. The reaction was monitored by TLC until complete conversion. After completion, the reaction mixture was poured into 525 mL of distilled water. The precipitate was then washed with cold methanol and dried under vacuum, providing **8** as yellow crystals (95.4 mg, 0.224 mmol, 79%). *R*_f_ = 0.41 (Cy/DCM 8:2); ^1^H NMR (300 MHz, CDCl_3_, δ): 1.75 (s, 9H, CH_3_), 7.56 (dd, ^3^*J* = 9.0 Hz, ^4^*J* = 2.0 Hz, 2H, CH), 7.99 (d, ^4^*J* = 2.0 Hz, 2H, CH), 8.14 (d, ^3^*J* = 9.0 Hz, 2H, CH); ^13^C NMR (75 MHz, CDCl_3_, δ): 28.5 (3C), 85.0 (1C), 116.5 (2C), 118.0 (2C), 122.7 (2C), 126.4 (2C), 130.6 (2C), 137.6 (2C), 150.6 (1C).

*N-Boc-3,6-bisallylcarbazole* (**9**), Same procedure than 3-allylcarbazole (**4a**), starting from 3,6-Dibromocarbazole-Boc (100.0 mg, 0.233 mmol, 1 eq.), PdCl_2_ (2.9 mg, 7.0 mol%) and PPh_3_ (17.3 mg, 28.0 mol%) and Allyltributylstannane (179 μL, 0.559 mmol, 2.4 eq.), providing **9** (70.0 mg, 0.201 mmol, 79%). *R*_f_ = 0.41 (Cy/DCM 8:2); ^1^H NMR (300 MHz, CDCl_3_, δ): 1.75 (s, 9H, CH_3_), 7.56 (dd, ^3^*J* = 9.0 Hz, ^4^*J* = 2.0 Hz, 2H, CH), 7.99 (d, ^4^*J* = 2.0 Hz, 2H, CH), 8.14 (d, ^3^*J* = 9.0 Hz, 2H, CH); ^13^C NMR (75 MHz, CDCl_3_, δ): 28.5 (3C), 85.0 (1C), 116.5 (2C), 118.0 (2C), 122.7 (2C), 126.4 (2C), 130.6 (2C), 137.6 (2C), 150.6 (1C).

*4,4′-Bis-(3,6-bisallylcarbazole)-1,1′-biphenyl* (**13a**), 4,4′-dibromobiphenyl (230.0 mg, 0.722 mmol, 1 eq.), 3,6-bisallylcarbazole (402.0 mg, 1.628 mmol, 2.3 eq.), CuI (83.4 mg, 0.433 mmol, 0.6 eq.), l-Proline (100.8 mg, 0.867 mmol, 1.2 eq.) and potassium carbonate (262.2 mg, 1.878 mmol, 2.6 eq.) were added to a previously dried reaction tube containing a magnetic bar. The tube was purged with argon for three cycles using Schlenk technique. The mixture was dissolved in 2.4 mL of dried DMSO and stirred for 18 h under argon at 100 °C. Finally, the reaction was cooled down to room temperature, quenched with distillated water, and stirred for 5 min. The organic layer was extracted with DCM (2x) and dried over magnesium sulphate. The volatiles were removed under reduced pressure, and the residue was purified by flask chromatography (cyclohexane/DMC 8:2), providing **13a** (136.0 mg, 0.205 mmol, 29%). *R*_f_ = 0.36 (Cy/DCM 8:2); ^1^H NMR (300 MHz, CDCl_3_, δ): 3.47 (d, ^3^*J* = 6.5 Hz, 8H, CH_2_), 4.98–5.06 (m, 8H, CH_2_), 5.98 (ddt, ^3^*J*_trans_ = 13.5 Hz, ^3^*J*_cis_ = 10.0 Hz, ^3^*J* = 6.5 Hz, 4H, CH), 7.13 (d, ^3^*J* = 8.5Hz, 4H, CH), 7.29 (d, ^3^*J* = 8.5 Hz, 4H, CH), 7.51 (d, ^3^*J* = 8.5 Hz, 4H, CH), 7.70 (d, ^3^*J* = 8.5 Hz, 4H, CH), 7.85 (s, 4H, CH); ^13^C NMR (75 MHz, CDCl_3_, δ): 40.6, 110.0, 115.8, 120.4, 124.0, 127.2, 127.5, 128.7, 132.0, 137.8, 138.7, 139.2, 140.0; MALDI (TOF): *m*/*z* calcd for C_48_H_40_N_2_: 644.319 [M]^+^; found: 644.467.

*4,4′-Bis-(3-allylcarbazole)-1,1′-biphenyl* (**13b**), Same procedure as 4,4′-bis-(3,6-bisallylcarbazole)-1,1′-biphenyl (**13a**), but starting from 4,4′-dibromobiphenyl (230.0 mg, 0.722 mmol, 1 eq.), 3-allylcarbazole (347.9 mg, 1.662 mmol, 2.3 eq.), CuI (83.4 mg, 0.433 mmol, 0.6 eq.), l-Proline (100.8 mg, 0.867 mmol, 1.2 eq.) and potassium carbonate (262.2 mg, 1.878 mmol, 2.6 eq.). Purified by silica gel column chromatography using cyclohexane/DCM (8:2 *v*/*v*), providing **13b** (144.0 mg, 0.255 mmol, 36%). *R*_f_ = 0.36 (Cy/DCM 8:2); ^1^H NMR (300 MHz, CDCl_3_, δ): 3.65 (d, ^3^*J* = 6.5 Hz, 4H, CH_2_), 5.15–5.25 (m, 4H, CH), 6.15 (ddt, ^3^*J*_trans_ = 16.5 Hz, ^3^*J*_cis_ = 10.0 Hz, ^3^*J* = 6.5 Hz, 2H, CH), 7.31–7.37 (m, 4H, CH), 7.47–7.50 (m, 4H, CH), 7.53–7.56 (m, 2H, CH), 7.70 (d, ^3^*J* = 8.5 Hz, 4H, CH), 7.90 (d, ^3^*J* = 8.5 Hz, 4H, CH), 8.03 (s, 2H, CH), 8.20 (d, ^3^*J* = 7.5 Hz, 2H, CH); ^13^C NMR (75 MHz, CDCl_3_, δ): 40.6, 110.1, 115.8, 120.3, 120.7, 123.8, 124.1, 126.3, 127.3, 127.7, 128.77, 132.2, 137.7, 138.7, 139.4, 139.8, 141.4.

*4,4′-(bis(3-3-(1,1,3,3,5,5,5-heptamethyltrisiloxane)propyl)carbazole)-1,1′-biphenyl* (**14a, CBP-2Si_3_**), 4,4′-bis(3-allylcarbazole)-1,1′-biphenyl **13a** (40.0 mg, 0.071 mmol, 1 eq.) was added to a previously dried reaction tube containing a magnetic bar. Toluene (5.0 mL) and 1,1,1,3,3,5,5-heptamethyltrisiloxane (76 μL, 0.280 mmol, 4 eq.) were added, and the mixture was purged with oxygen for 5 min. Then, the Karstedt’s catalyst (50 ppm/SiH) was finally introduced. The mixture was stirred overnight at 90 °C under oxygen atmosphere. The volatiles were evaporated, and the crude product was purified by column chromatography on silica gel with petroleum ether/CH_2_Cl_2_ 8:2 as eluent providing the desired product **14a** as a white solid. *R*_f_ = 0.50 (Cy/DCM 7:3); ^1^H NMR (300 MHz, CDCl_3_, δ): 0.05–0.10 (m, 42H, CH_3_), 0.65–0.71 (m, 4H, CH_2_), 1.74–1.84 (m, 4H, CH_2_), 2.86 (t, ^3^*J* = 7.5 Hz, 4H, CH_2_), 7.25–7.33 (m, 4H, CH), 7.43 (t, ^3^*J* = 7.5 Hz, 4H, CH), 7.52 (d, ^3^*J* = 8.0 Hz, 2H, CH), 7.71 (d, ^3^*J* = 8.5 Hz, 4H, CH),7.91 (d, ^3^*J* = 8.5 Hz, 4H, CH), 7.97 (s, 2H, CH), 8.16 (d, ^3^*J* = 8.0 Hz, 2H, CH); MALDI (TOF): *m*/*z* calcd for C_56_H_76_N_2_O_4_Si_6_: 1008.442 [M]^+^; found: 1008.452.

*4,4′-(tetra(3-3-6-6-(1,1,3,3,5,5,5-heptamethyltrisiloxane) propyl)carbazole)-1,1′-biphenyl* (**14b, CBP-4Si_3_**), Same procedure as product (**14a**), starting from 4,4′-bis(3,6-bisallylcarbazole)-1,1′-biphenyl (40.0 mg, 0.062 mmol, 1 eq.), 1,1,1,3,3,5,5-heptamethyltrisiloxane (152 μL, 0.560 mmol, 8 eq.) and the Karstedt‘s catalyst (50 ppm/SiH), providing **14b** (72.0 mg, 0.047 mmol, 72%). Tg ~ −60 °C; ^1^H NMR (300 MHz, CDCl_3_, δ): 0.00 (br s, 24H, CH_3_), 0.05 (br s, 60H, CH_3_), 0.60–0.66 (m, 8H, CH_2_), 1.74–1.84 (m, 4H, CH_2_), 2.8 (t, ^3^*J* = 7.5 Hz, 8H, CH_2_), 7.21 (d, ^3^*J* = 7.0 Hz, 4H, CH), 7.39 (d, ^3^*J* = 8.5 Hz, 4H, CH), 7.66 (d, ^3^*J* = 8.5 Hz, 4H, CH), 7.85 (d, ^3^*J* = 8.5 Hz, 4H, CH), 7.90 (s, 4H, CH); ^13^C NMR (75 MHz, CDCl_3_, δ): 0.6 (12C), 1.7 (8C), 2.2 (8C), 18.5 (4C), 26.5 (4C), 40.1 (4C), 109.8 (4C), 120.1 (4C), 123.9 (4C), 127.0 (4C), 127.5 (4C), 127.7 (4C), 134.7 (4C), 138.0 (4C), 139.8 (4C), 139.8 (4C). MALDI (TOF): *m*/*z* calcd for C_77_H_130_N_2_O_8_Si_12_: 1532.6902 [M + H]^+^; found 1532.555.

## Figures and Tables

**Table 1:**
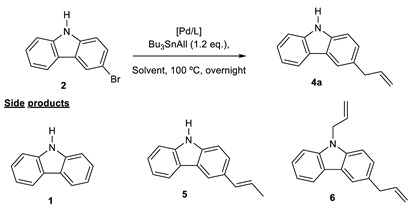
Optimization of the Stille coupling of 3-bromocarbazole.

Entry	Catalytic System	Solvent	1	4a	5	6
1	5.0 mol% Pd(PPh_3_)_4_	DMF	-	-	-	Mixture of N,3-bisallyl isomers
2	2.5 mol% PdCl_2_ 10.0 mol% PPh_3_	DMF	4%	74%	<1%	-
3	3.5 mol% PdCl_2_ 14.0 mol% PPh_3_	DMF	1%	98%	<1%	-
4	6.0 mol% PdCl_2_ 20.0 mol% PPh_3_	DMF	1%	91%	8%	-
5	3.5 mol% PdCl_2_ 14.0 mol% PPh_3_	Toluene	2%	77%	8%	-

Results obtained by GC-MS conversion.

## Data Availability

Data is available in this article and its Supplementary Materials.

## Supplementary Materials

The Supplementary Materials describing the mass and NMR characterization of the products are available online.

## Sample Availability

Not available.
