# Supplementary material for: Functionalization of Biphenylcarbazole (CBP) with Siloxane-Hybrid Chains for Solvent-Free Liquid Materials
Source: Molecules. 2021 Dec 24;27(1):89. doi: 10.3390/molecules27010089 (PMC8746609; doi:10.3390/molecules27010089)
Supplement: Supplementary file 1 [file molecules-27-00089-s001.zip › molecules-1486375-supplementary.pdf]

Supporting Information To:

Functionalization of biphenylcarbazole (CBP) with siloxane-hybrid chains for solvent-free liquid materials

Janah Shaya[1,2,3*], Gabriel Correia[1], Benoît Heinrich[1], Jean-Charles Ribierre[4], Kyriaki Polychronopoulou[5,6], Loïc Mager[1], Stéphane Méry[1*]

[1] CNRS-Strasbourg University, IPCMS UMR7504, 23 rue du Loess, BP 43, 67034 Strasbourg, Cedex, France

[2] Department of Chemistry, College of Arts and Sciences, Khalifa University, Abu Dhabi P.O. Box 127788, United Arab Emirates

[3] College of Medicine and Health Sciences, Khalifa University, Abu Dhabi P.O. Box 127788, United Arab Emirates;

[4] Center for Organic Photonics and Electronics Research (OPERA), Kyushu University, 744 Motooka, Nishi, Fukuoka 819-0395, Japan

[5] Department of Mechanical Engineering, Khalifa University of Science and Technology, Abu Dhabi,

P.O. Box 127788, UAE

[6] Center for Catalysis and Separation, Khalifa University of Science and Technology, Abu Dhabi,

P.O. Box 127788, UAE

* Corresponding authors

Table of contents

Pages

NMR and MALDI Methods........................S2

Characterization of product (2)........................S2

Characterization of product (3)........................S3

Characterization of product (4a)........................S3-4

Characterization of product (4b)........................S4

Characterization of product (7a)........................S5-6

Characterization of product (7b)........................S6-7

Characterization of product (8)........................S7-8

Characterization of product (9)........................S8-9

Characterization of product (13a)........................S9-10

Characterization of product (13b)........................S11

Characterization of product (14a)........................S12

Characterization of product (14b)........................S13-14

^{1}H and ^{13}C NMR spectra were recorded on a Bruker Avance 300 and a Bruker 400 Ultrashield™ NMR spectrometers, with an internal lock on the 2H-signal of the solvent. Chemical shifts (δ) are given in ppm to the nearest 0.01 (^{1}H) or 0.1 ppm (^{13}C{^{1}H}NMR) (recorded with complete proton decoupling and written as ^{13}C in the experimental part for simplicity). The coupling constants (J) are given in Hertz (Hz). The signals are reported as follows: (s = singlet, d = doublet, t = triplet, quint = quintet, m = multiplet). Mass spectra analyses were performed by using a Maldi-TOF or GC/MS. The former were carried out on a time-of-flight mass spectrometer (MALDI-TOF-TOF Autoflex II TOF-TOF, Bruker Daltonics, Bremen, Germany) equipped with a nitrogen laser (λ = 337 nm). The latter was carried out on a gas chromatograph coupled with an electron ionization mass spectrometer 7090-5975C from Agilent Technologies.

<u>^{1}H NMR of (2)</u>

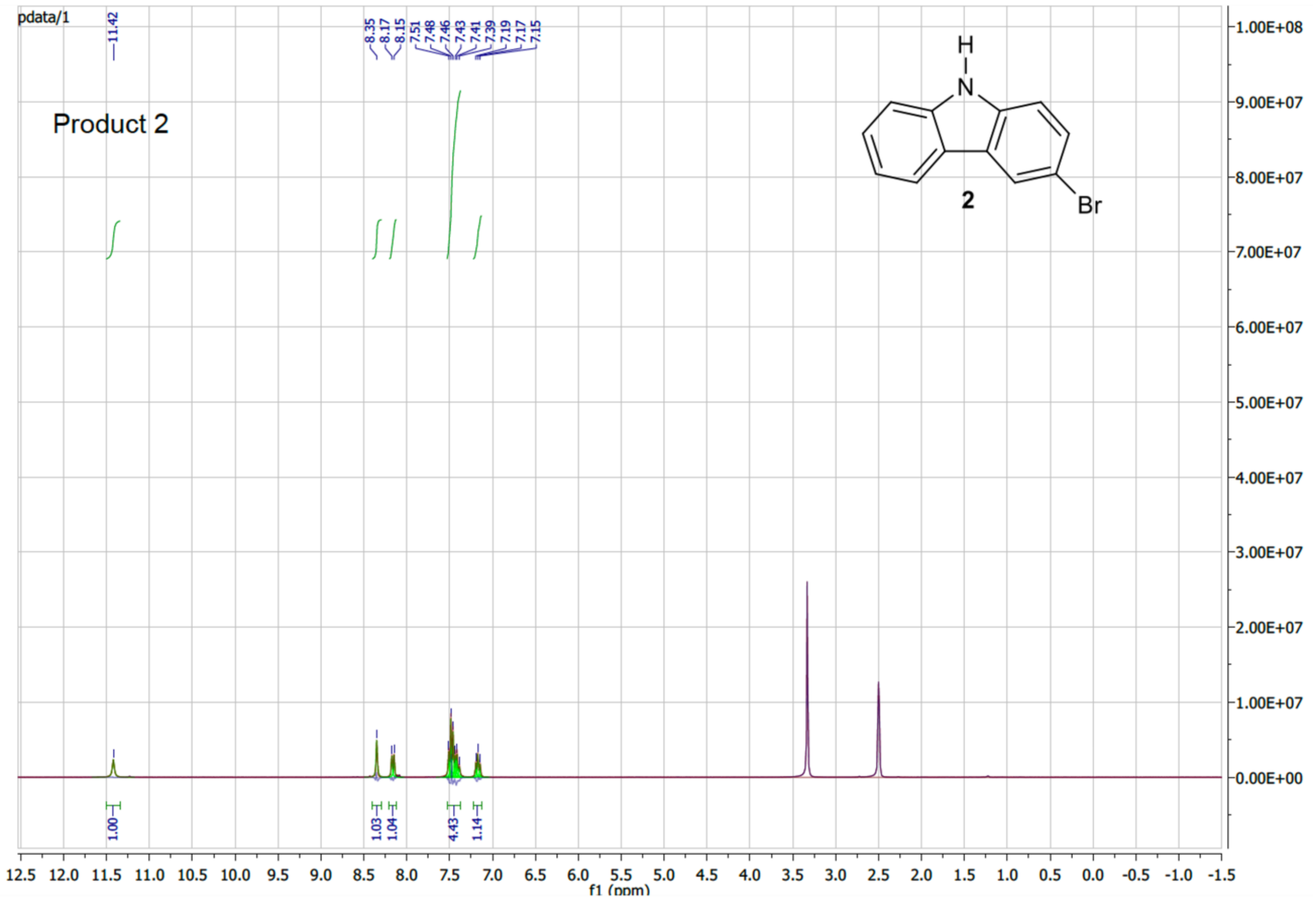

^{1}H NMR of (3)

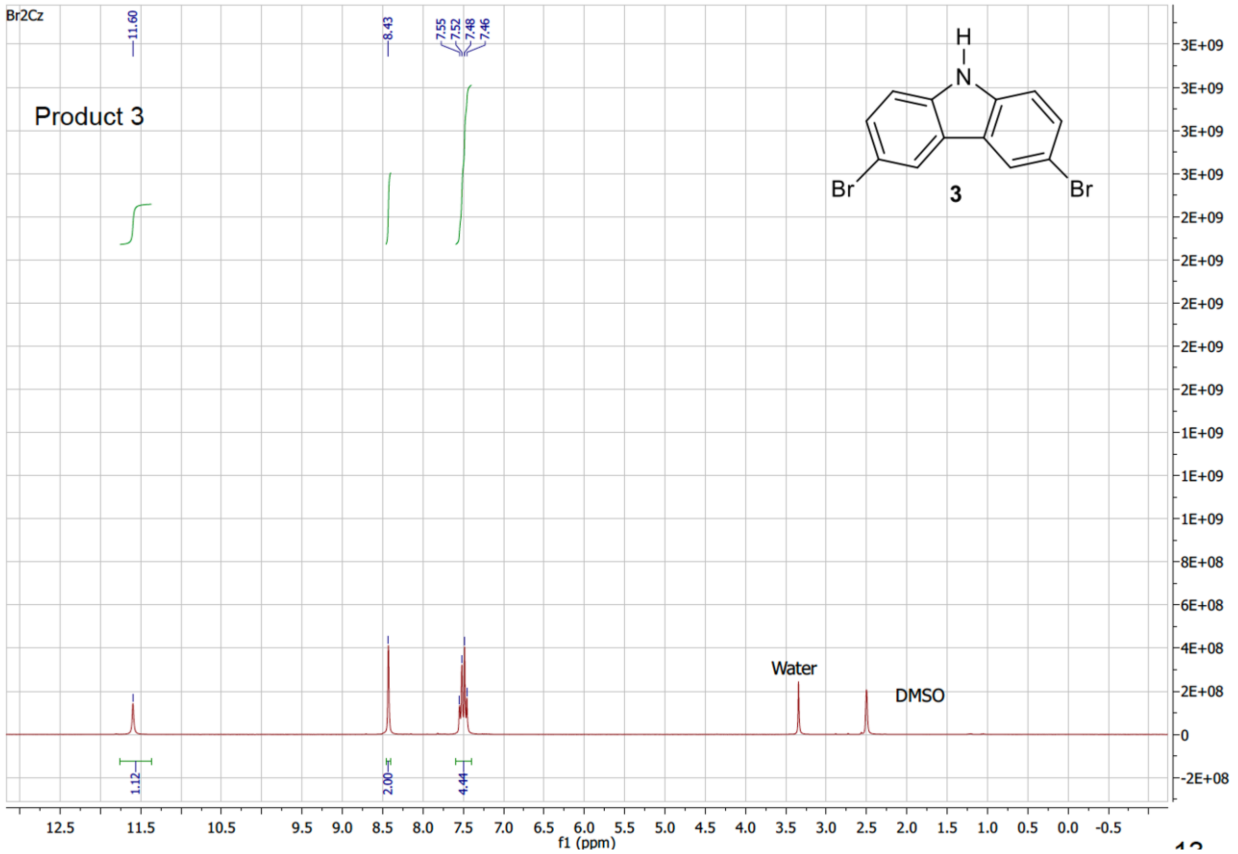

^{1}H NMR of (4a)

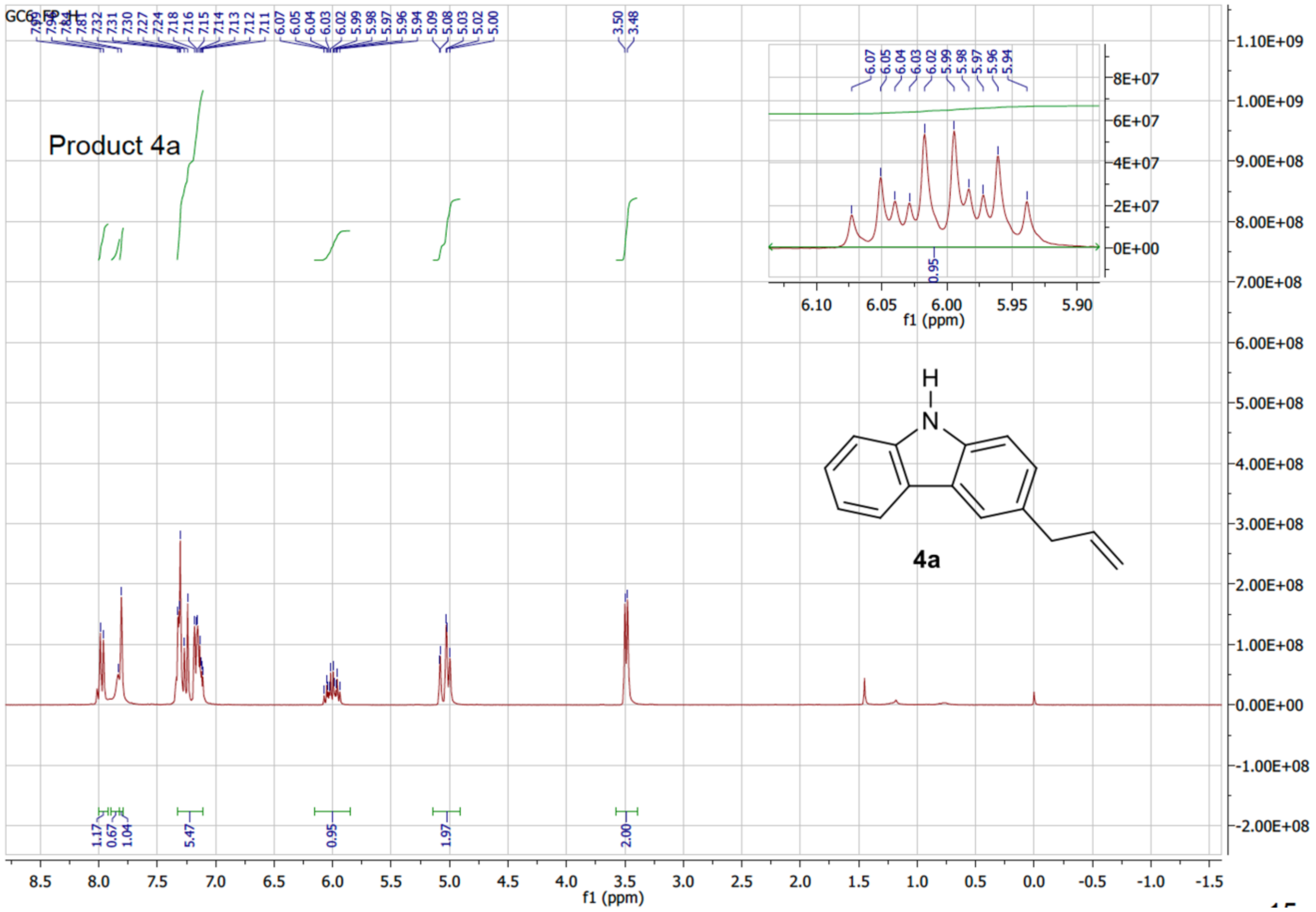

^{13}C NMR of (4a)

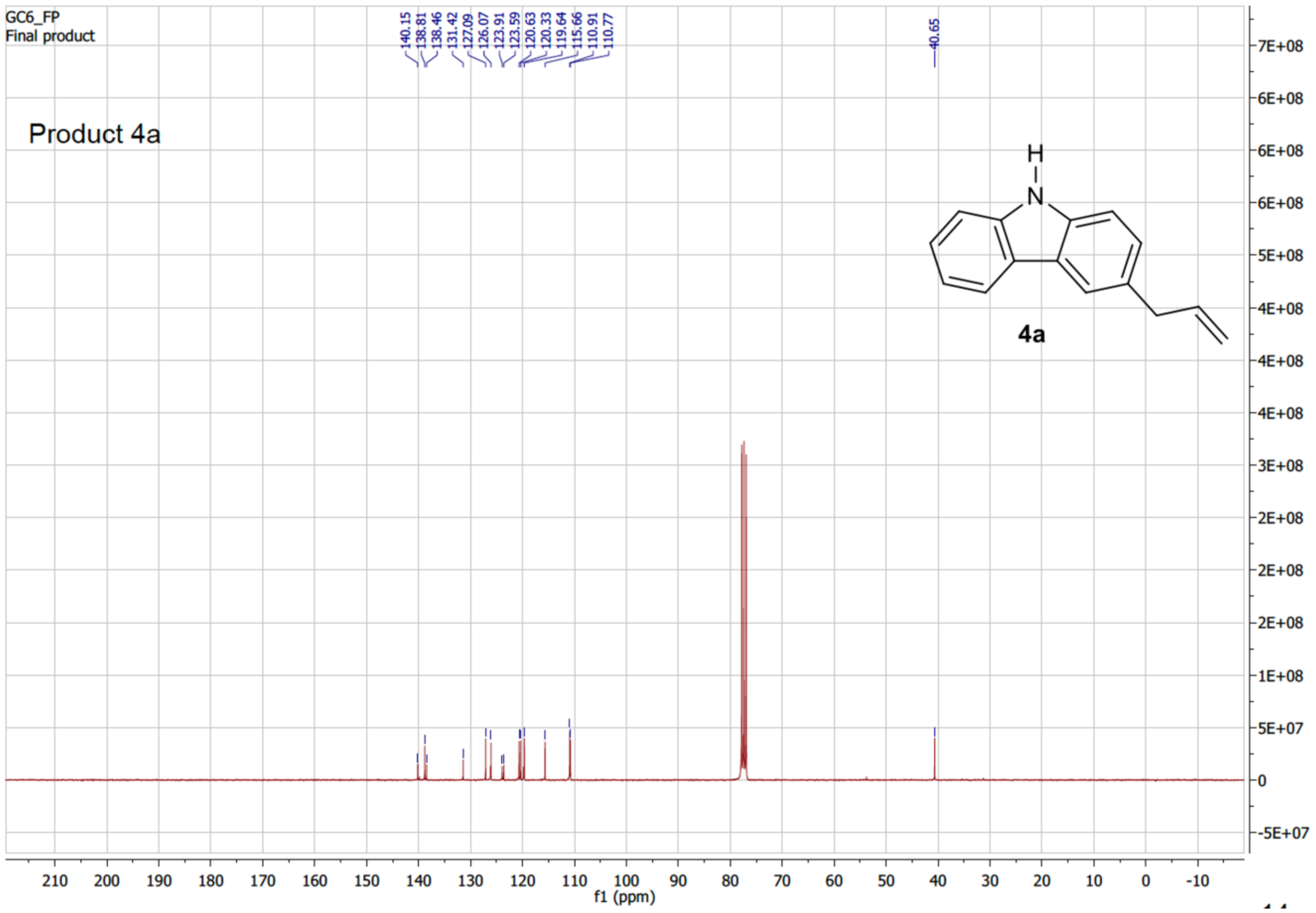

^{1}H NMR of (4b)

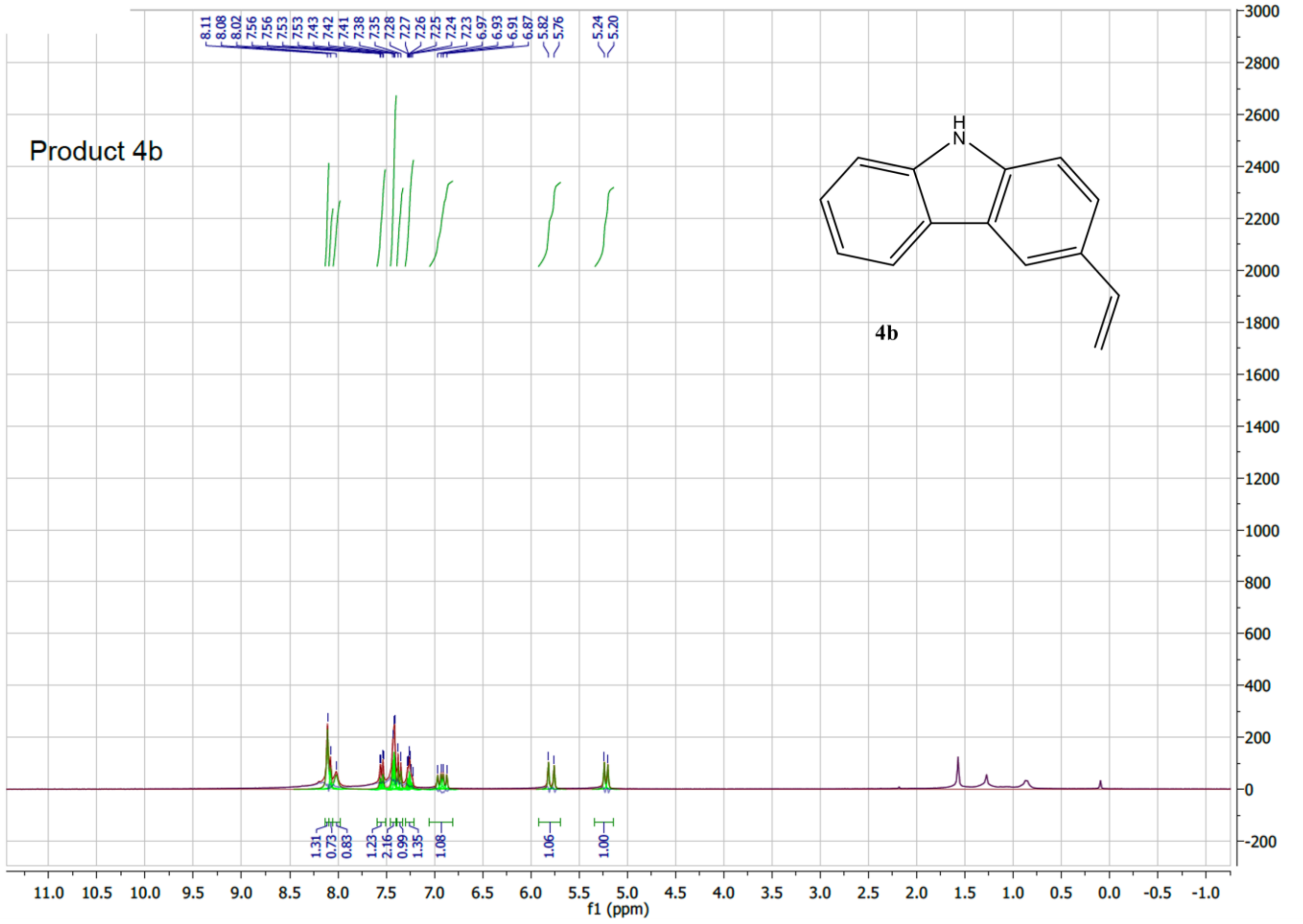

^{1}H NMR of (7a)

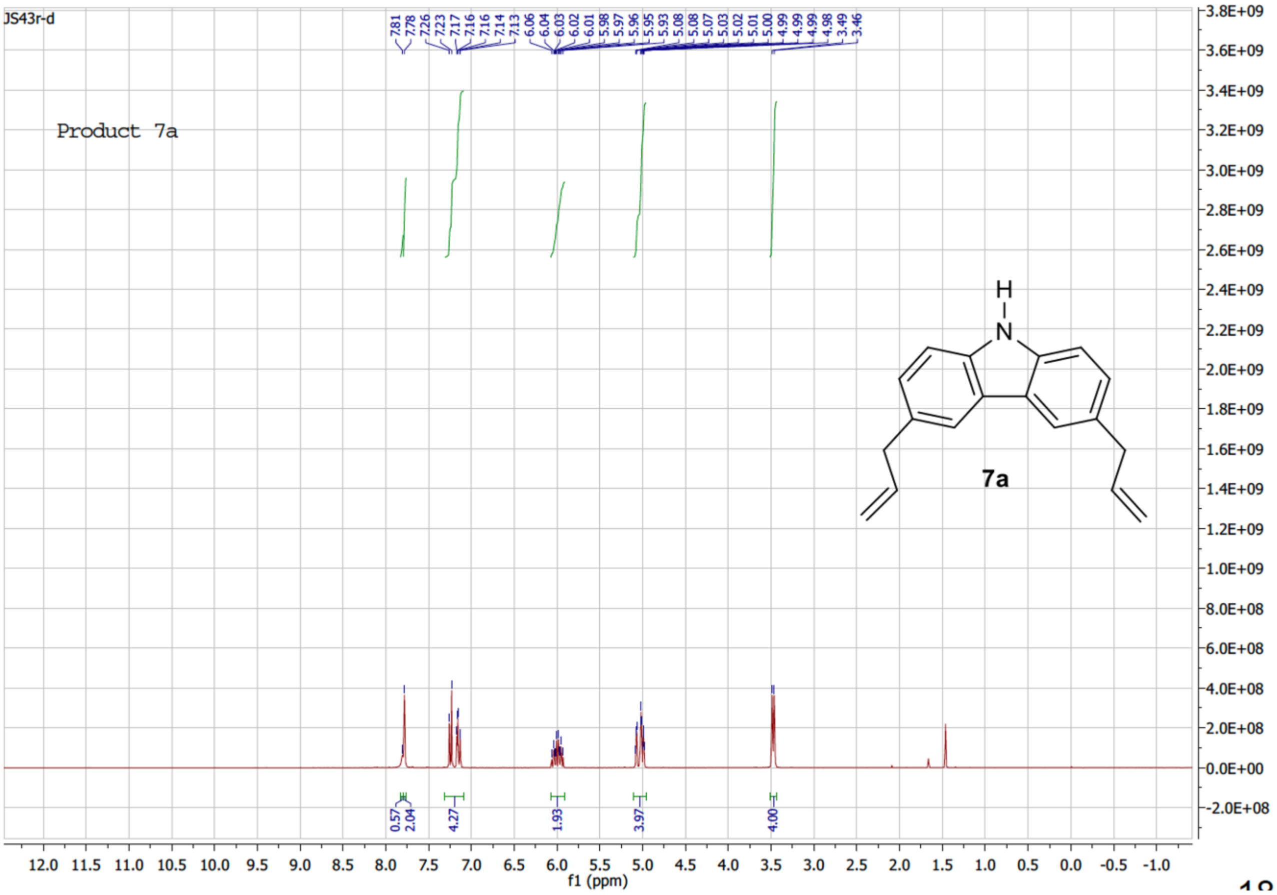

^{13}C NMR of (7a)

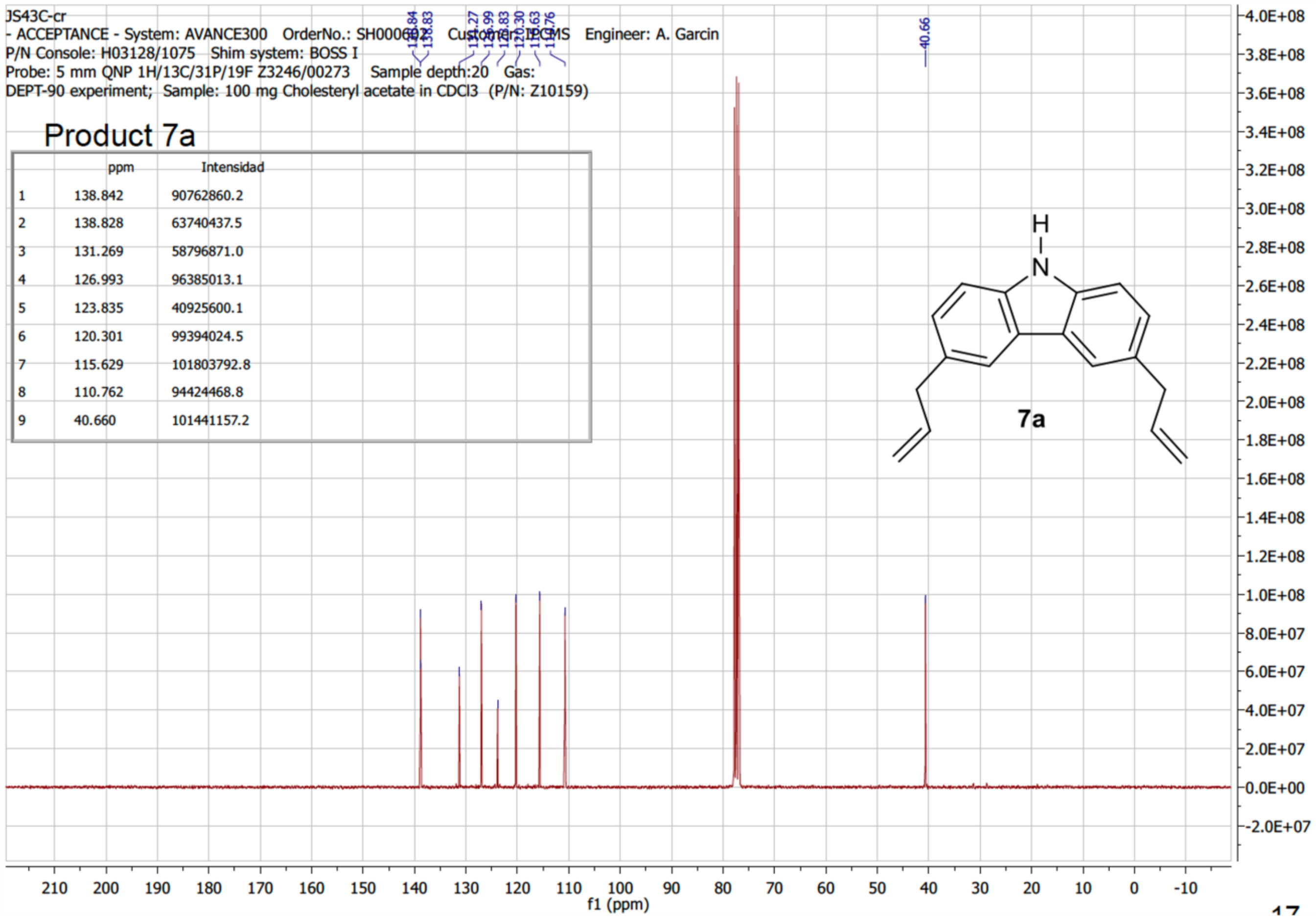

MALDI of (7a)

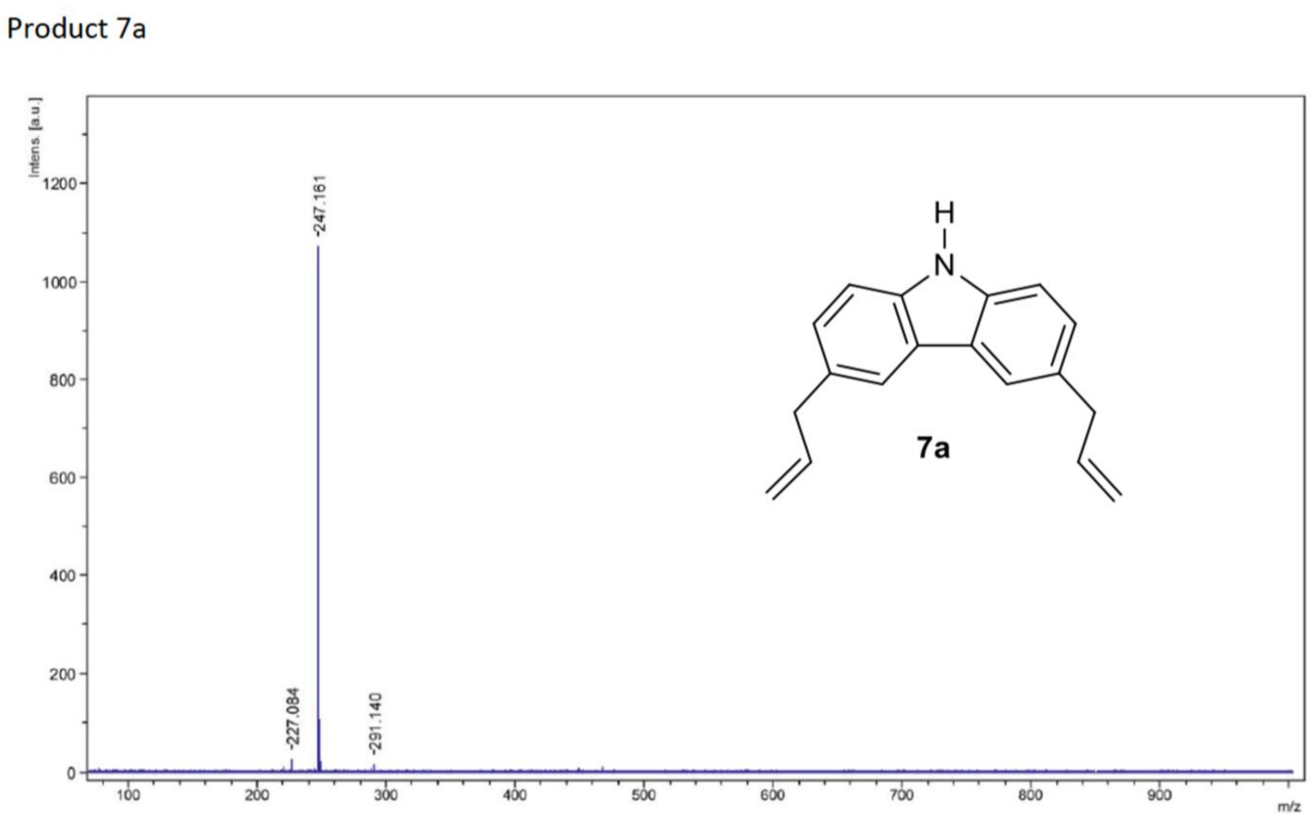

^{1}H NMR of (7b)

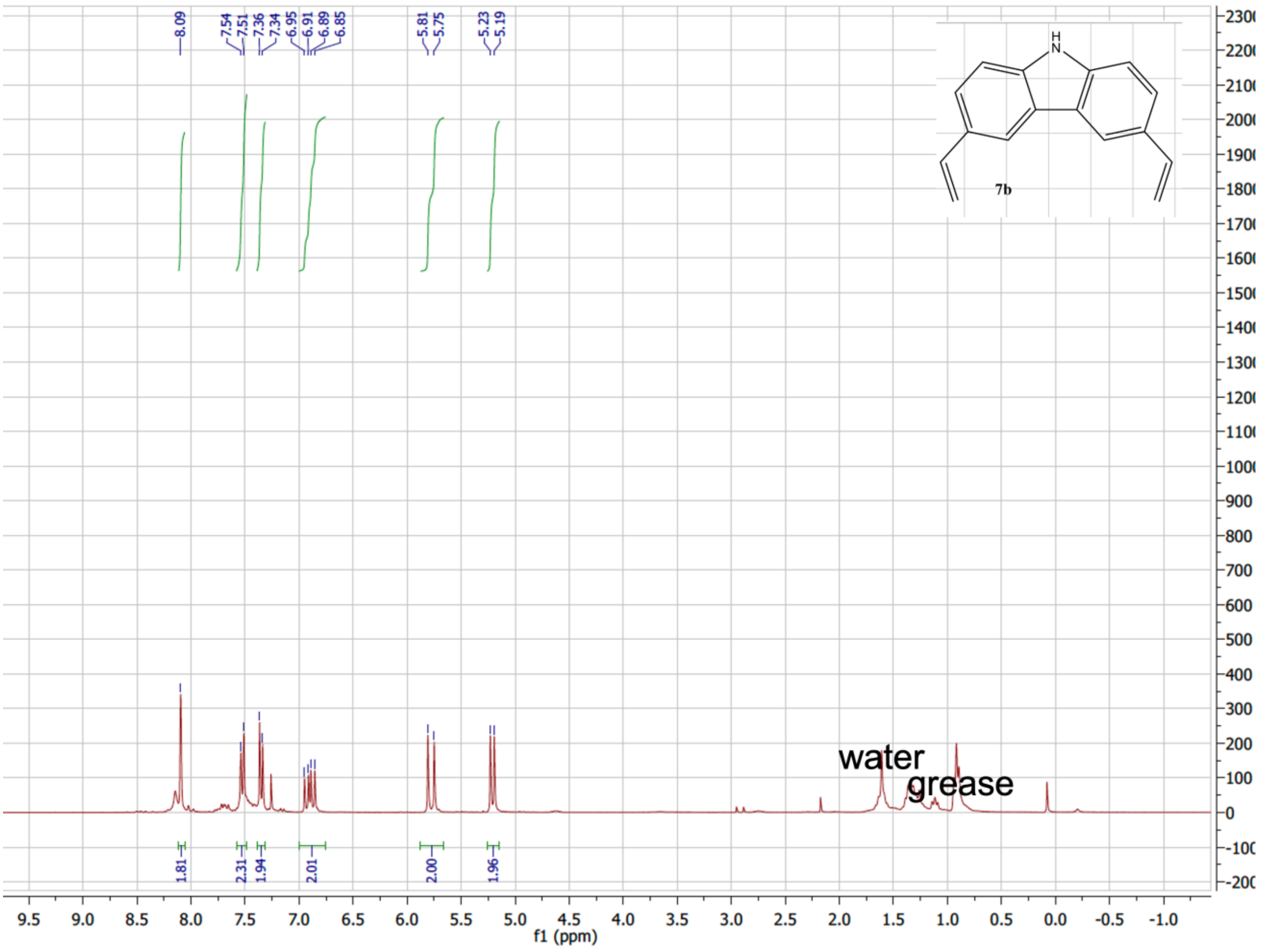

^{13}C NMR of (7b)

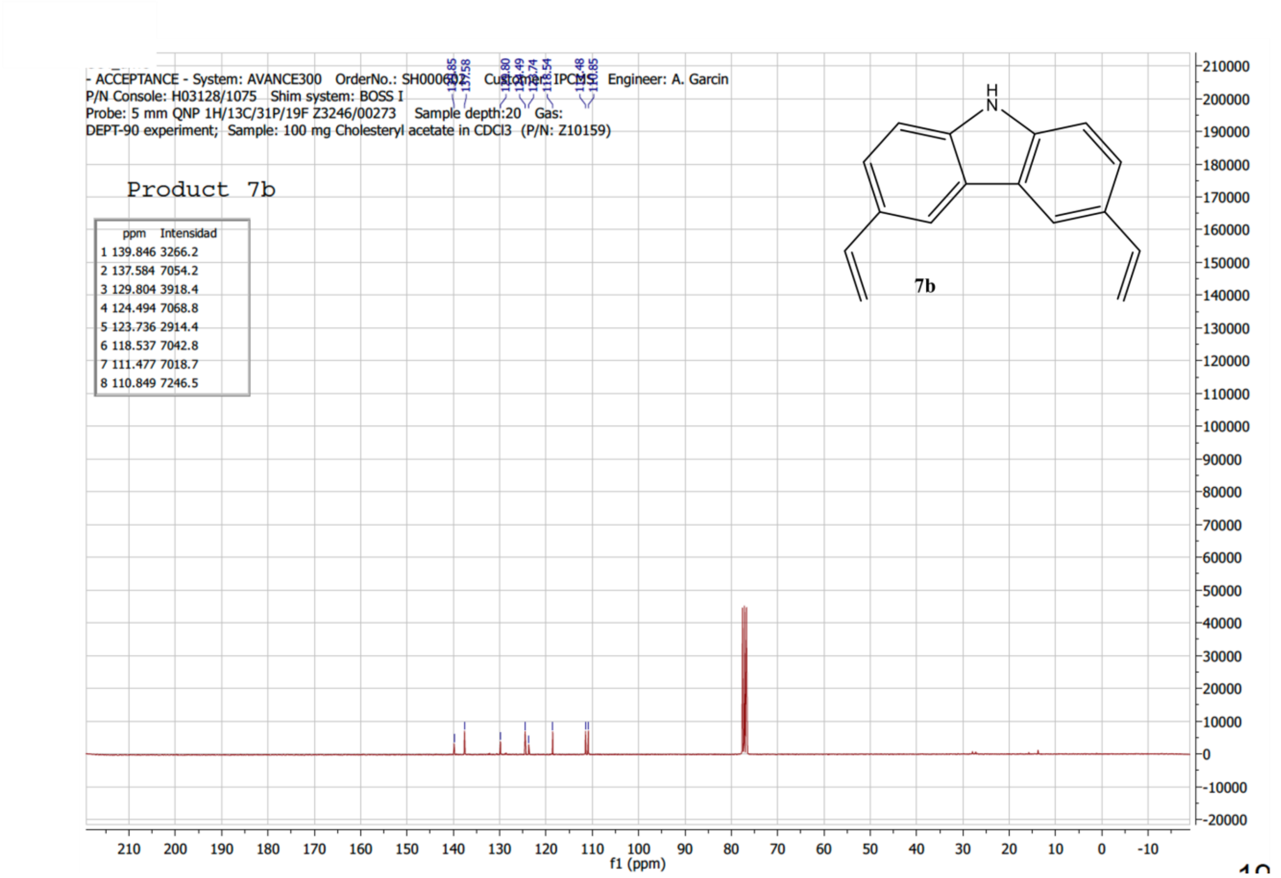

^{1}H NMR of (8)

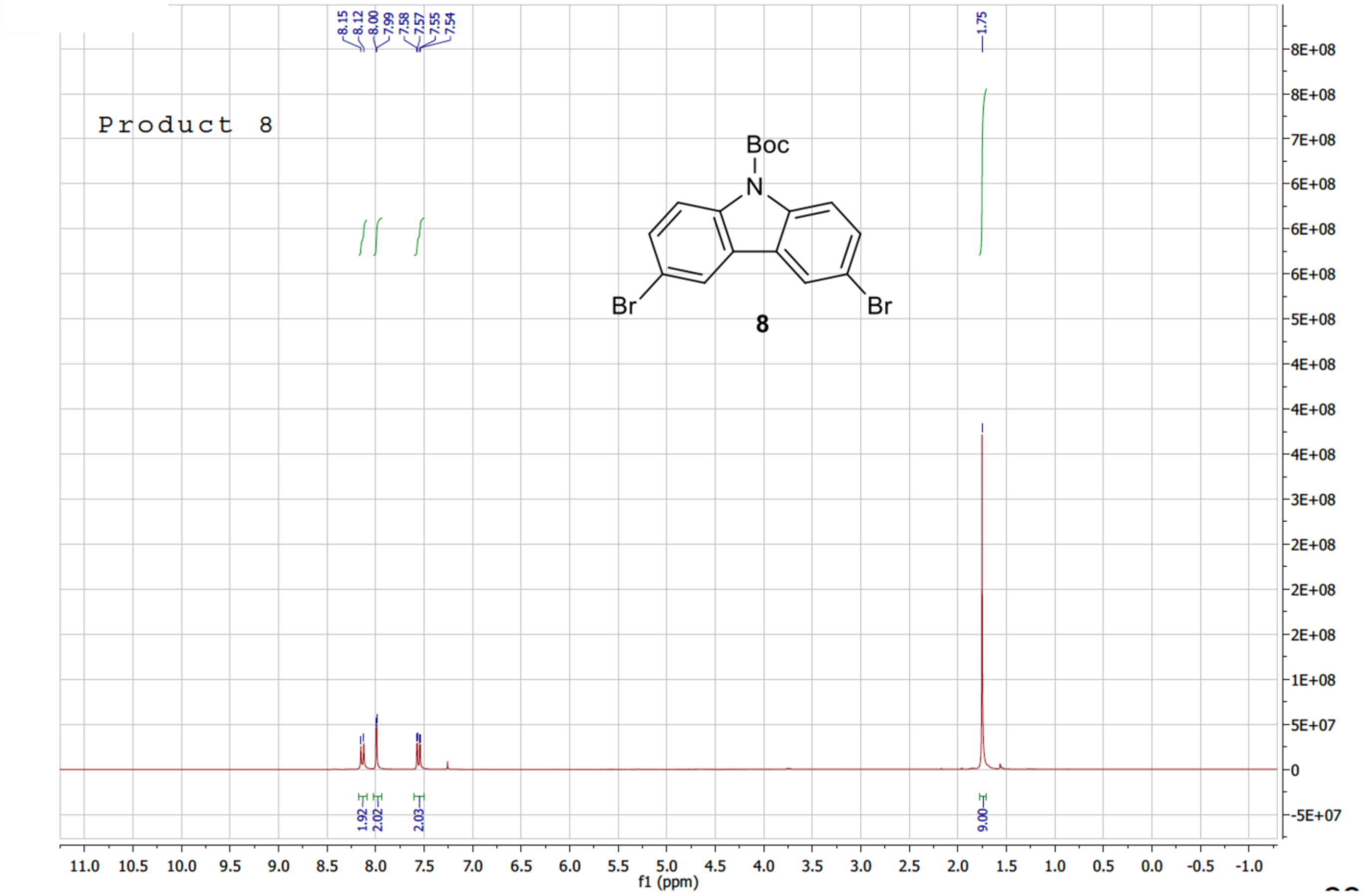

^{13}C NMR of (8)

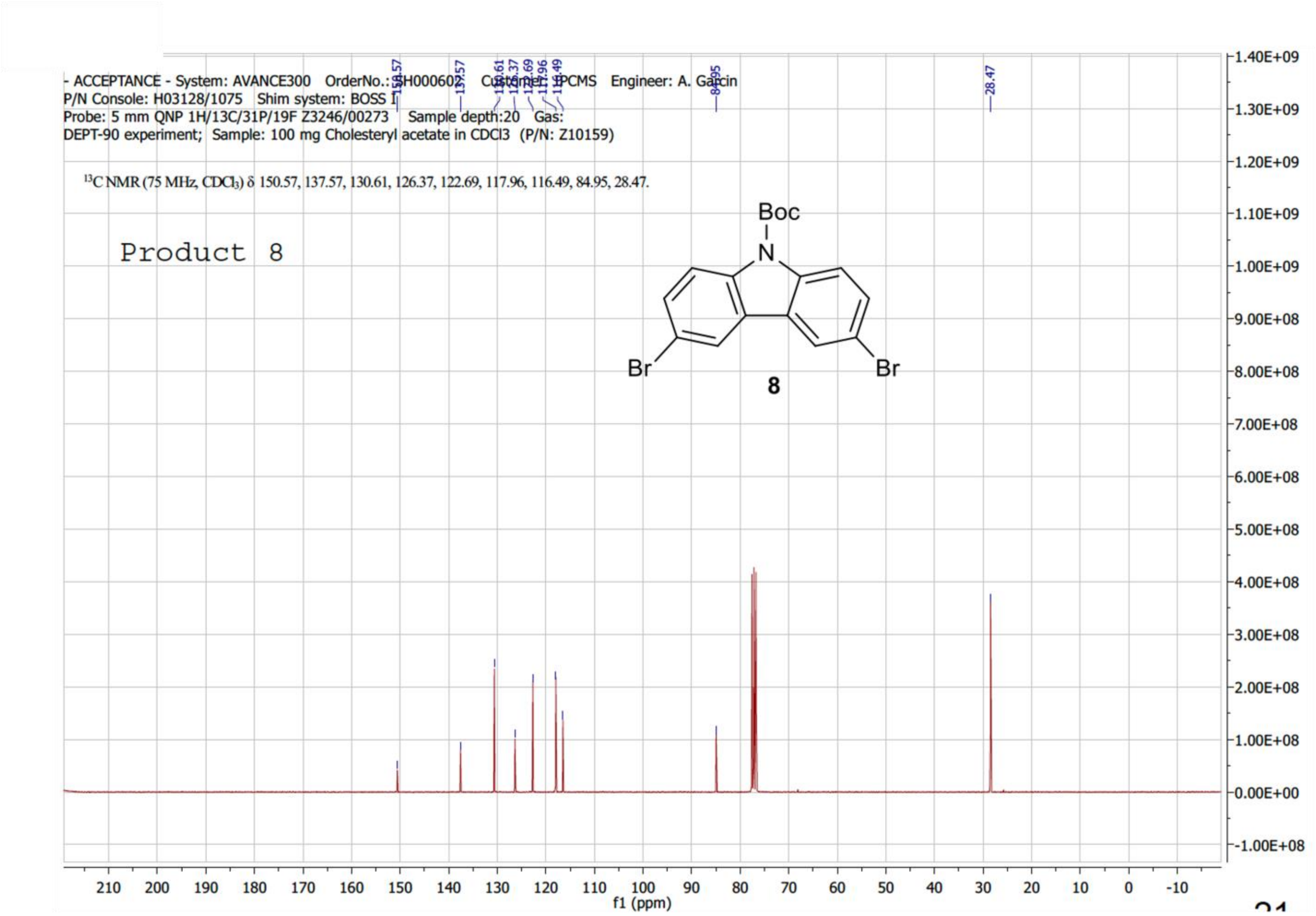

^{1}H NMR of (9)

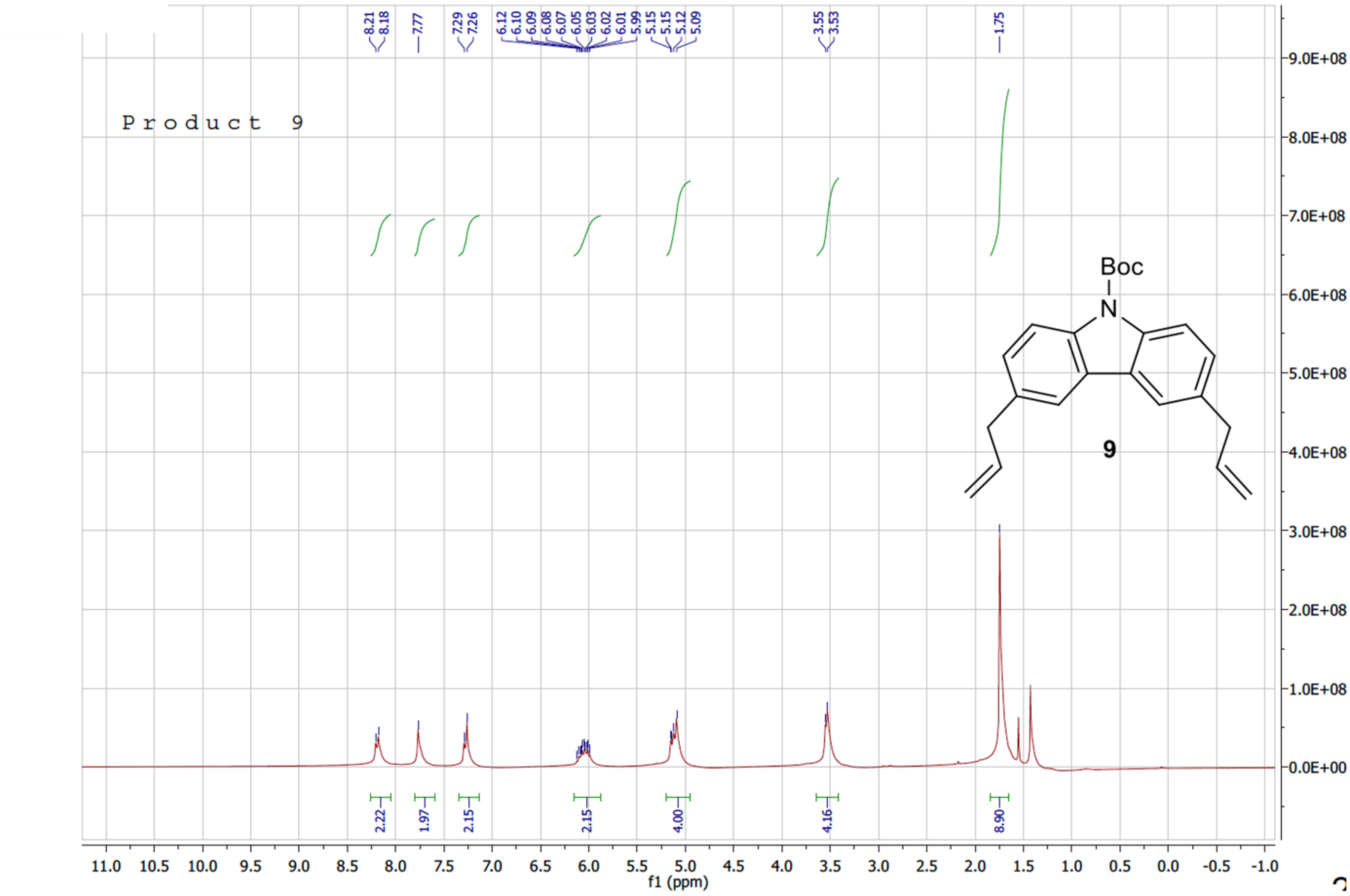

13C NMR of (9)

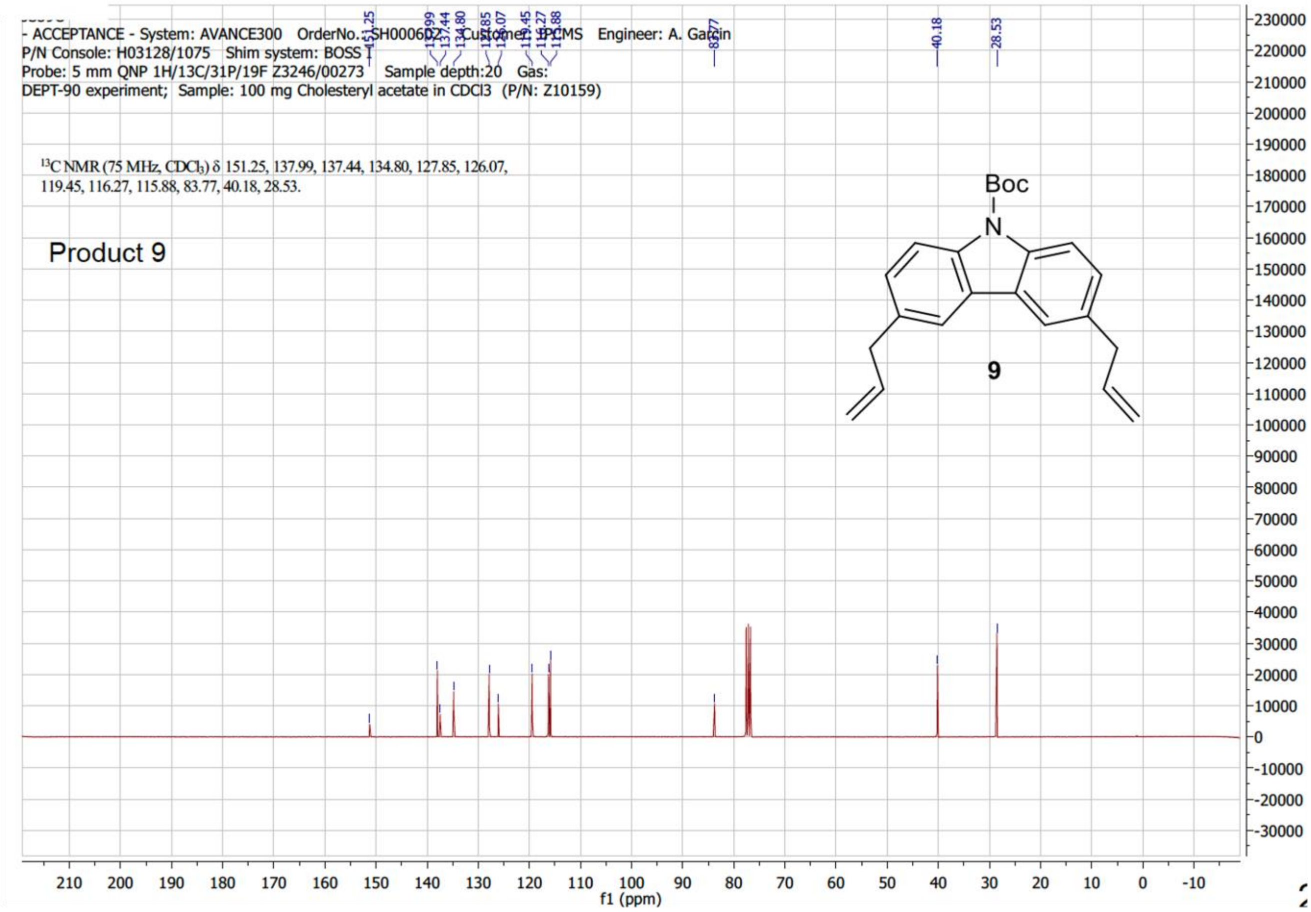

1H NMR of (13a)

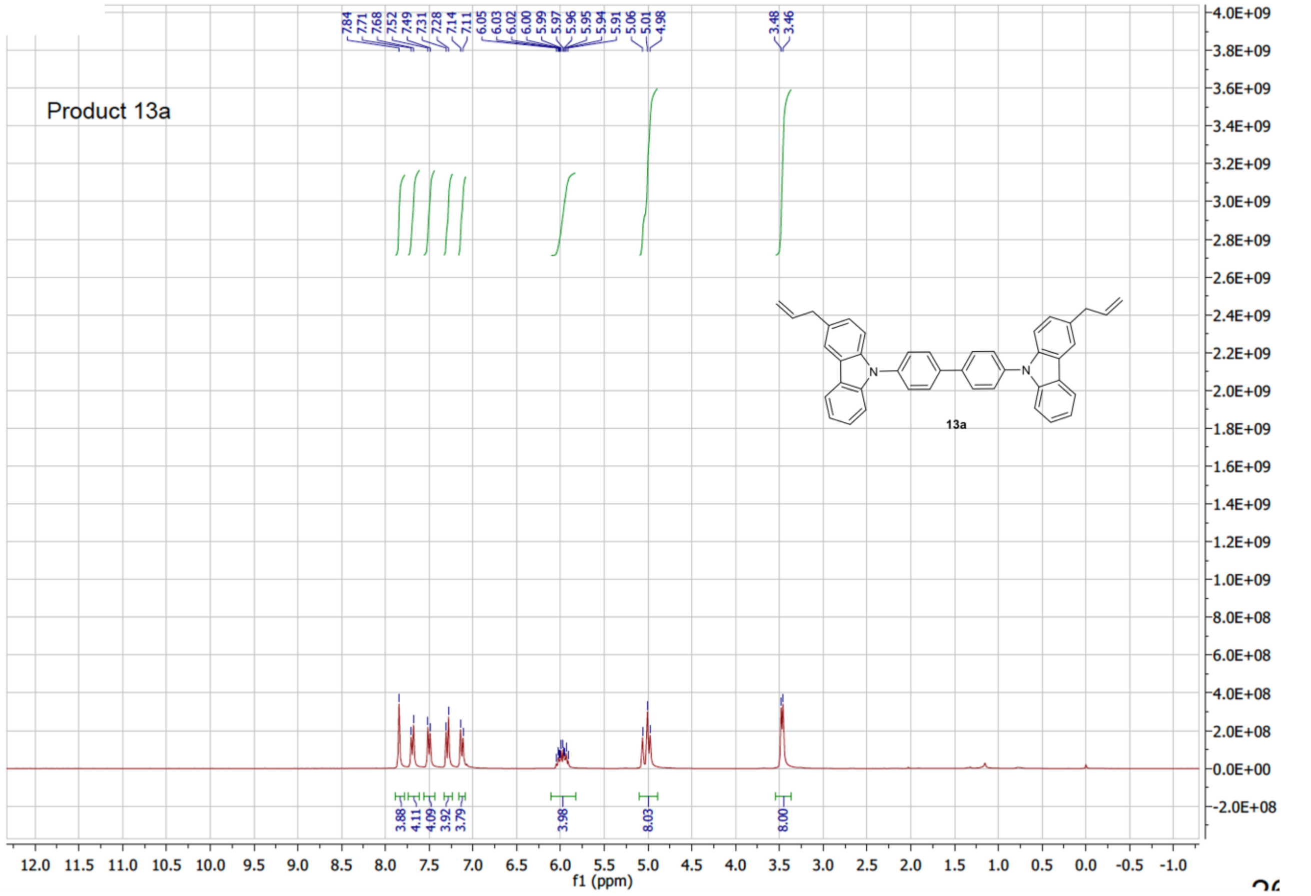

^{13}C NMR of (13a)

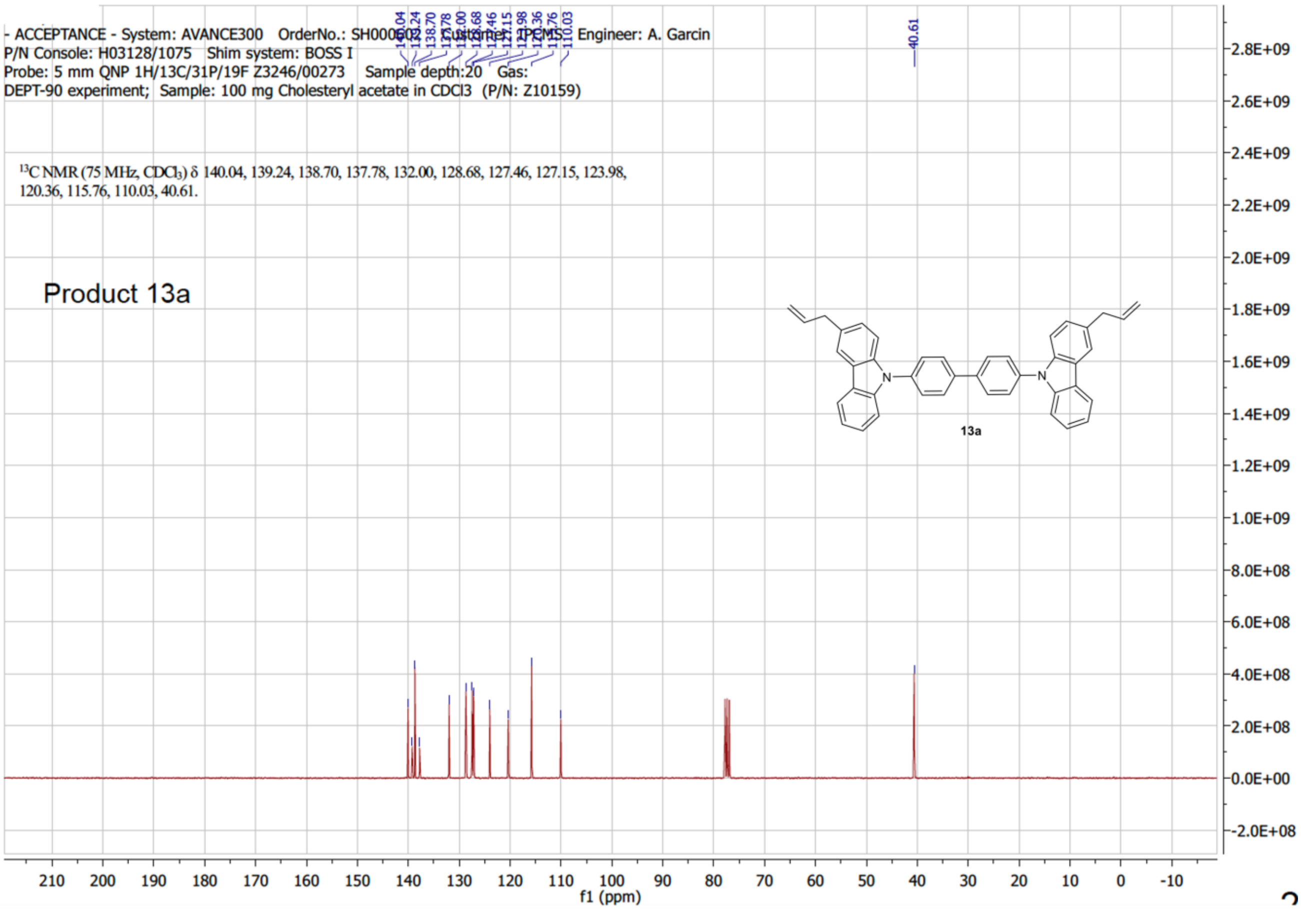

MALDI of (13a)

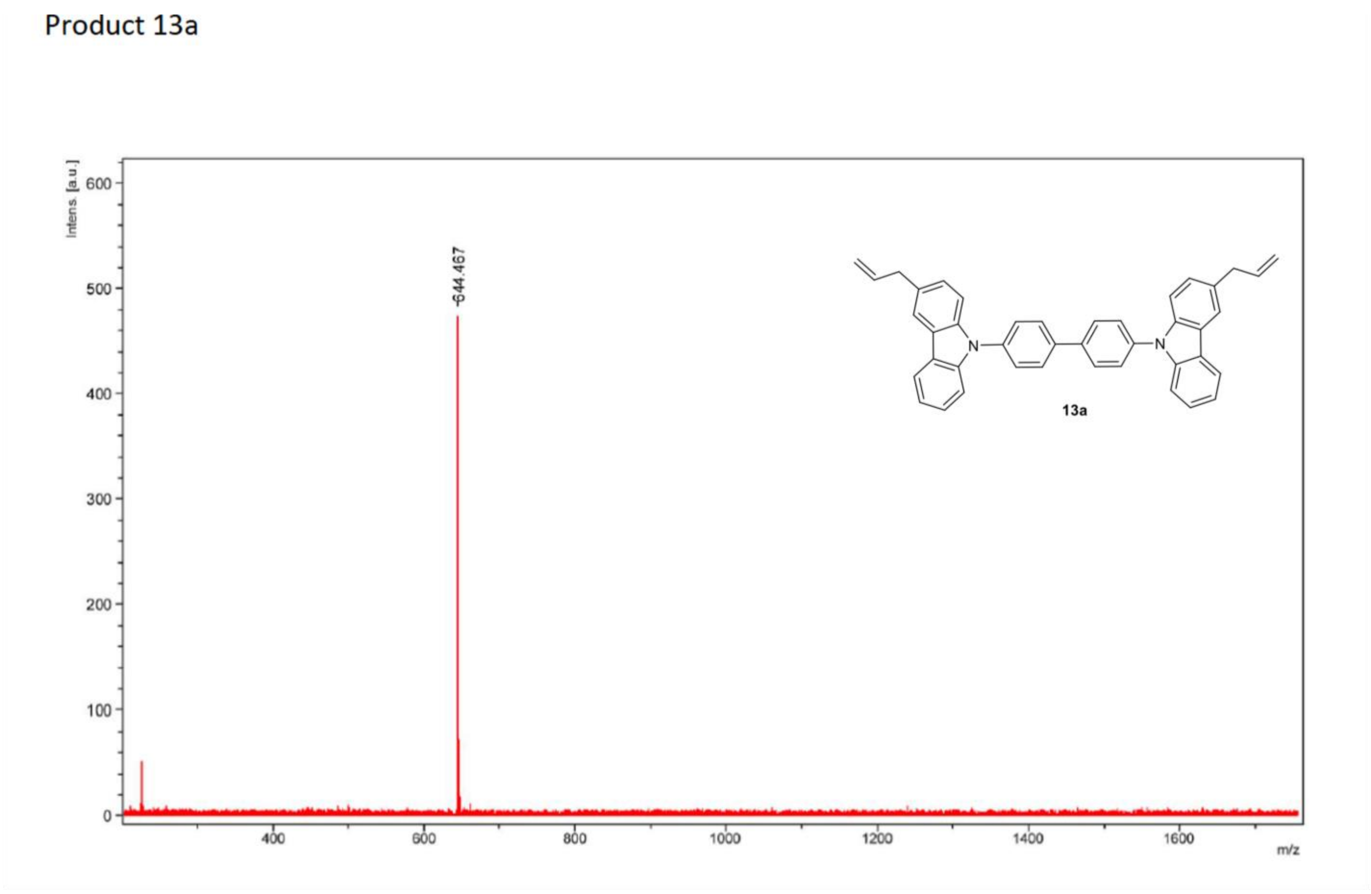

^{1}H NMR of (13b)

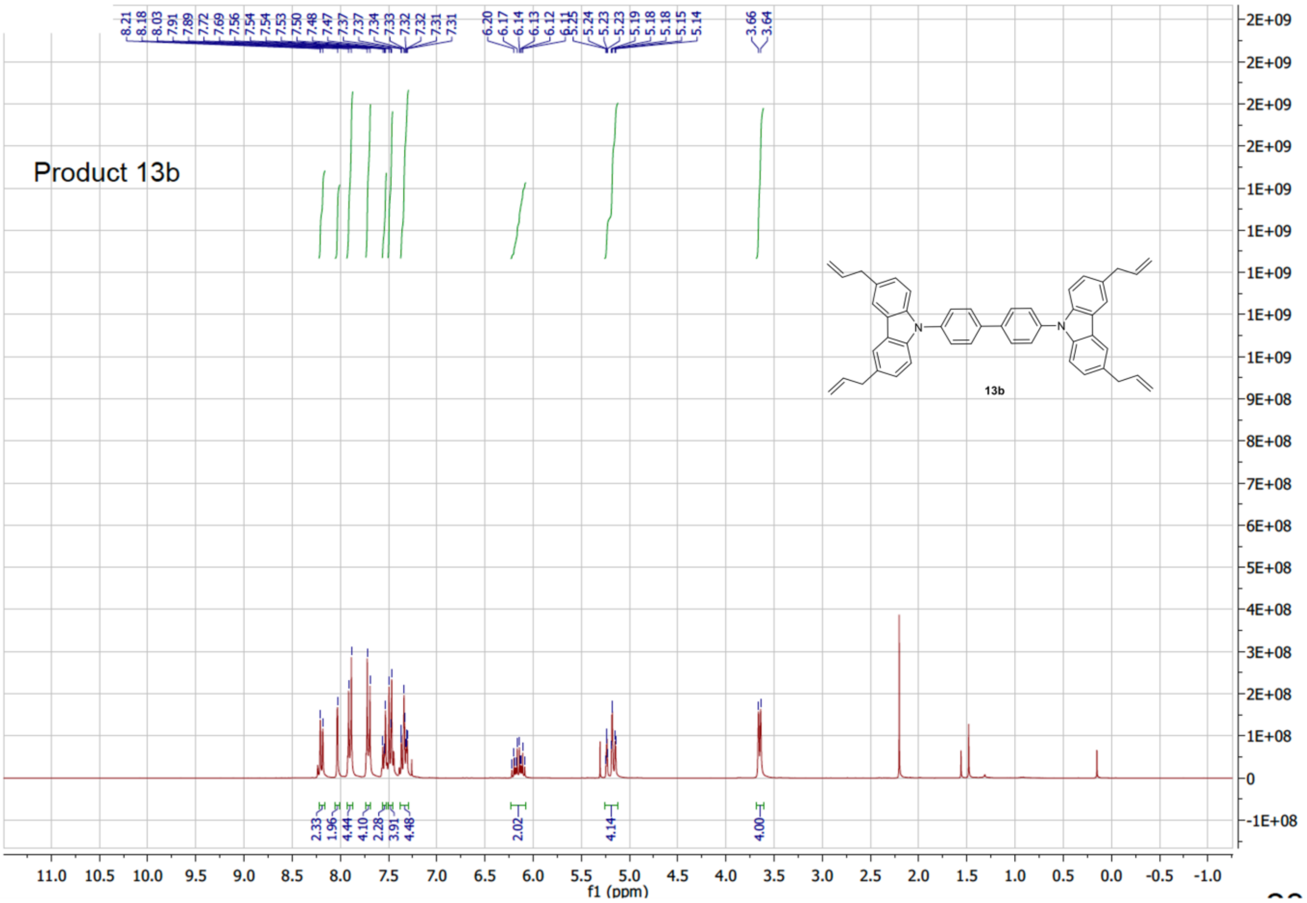

^{13}C NMR of (13b)

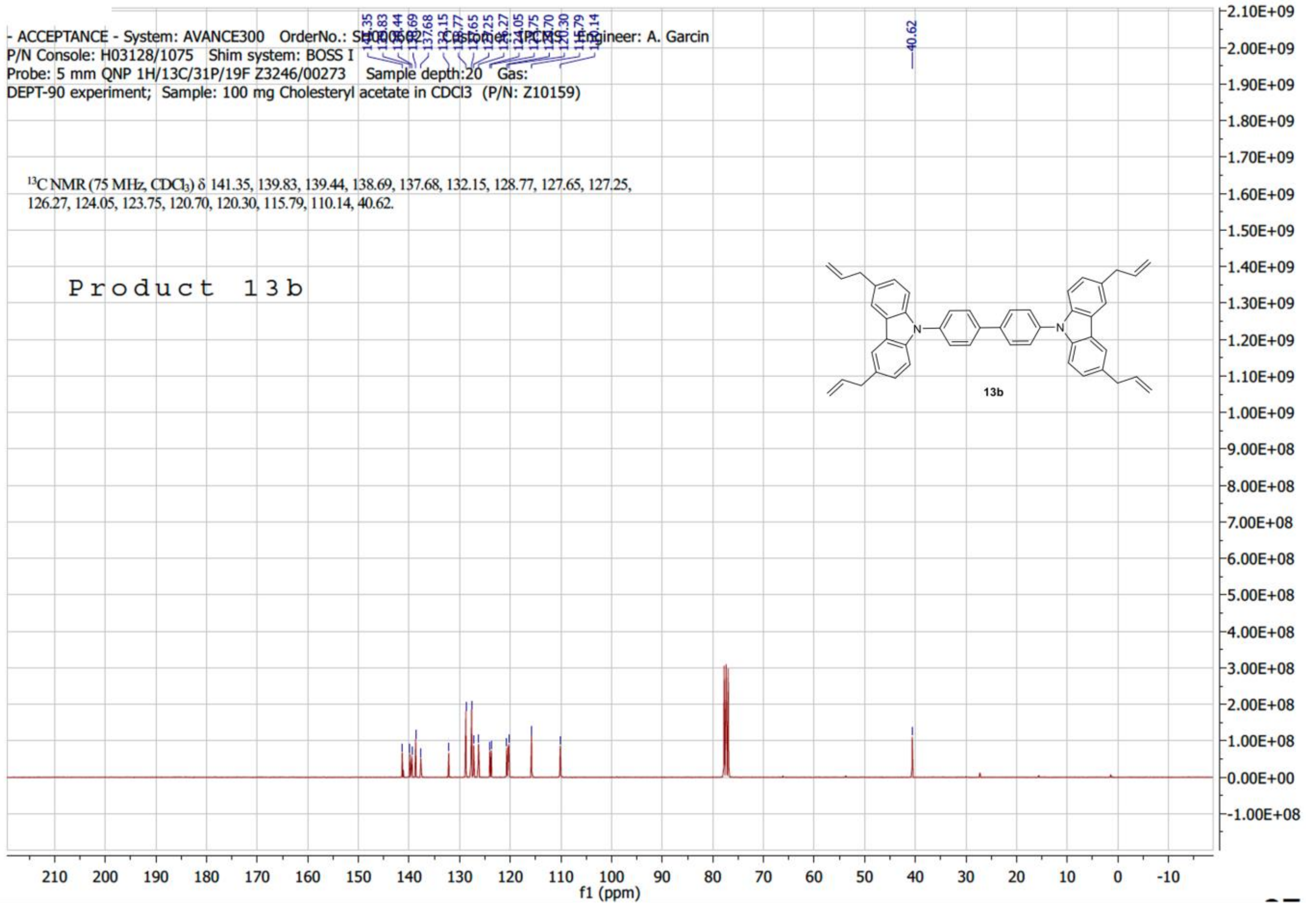

^{1}H NMR of (14a)

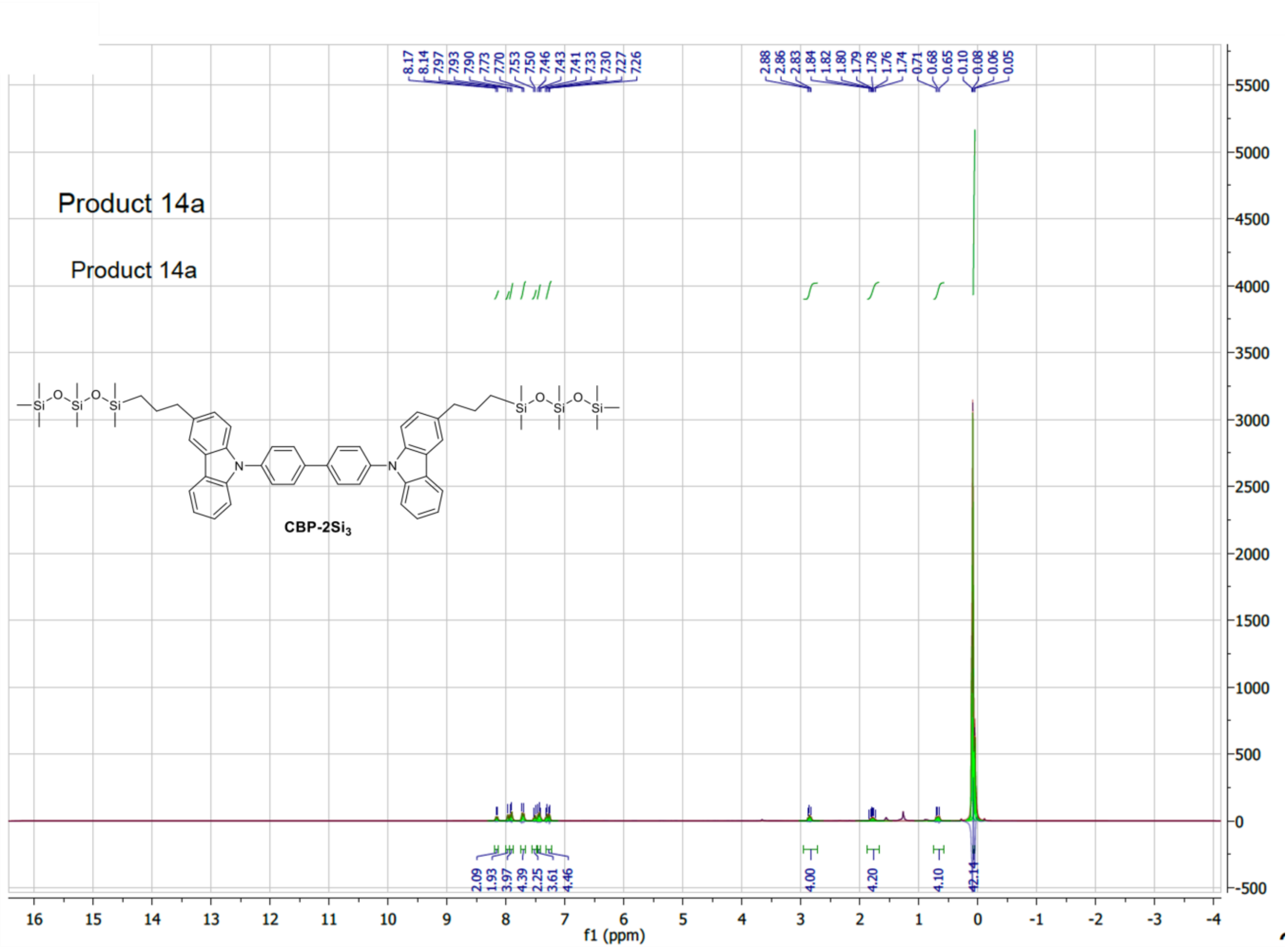

MALDI of (14a)

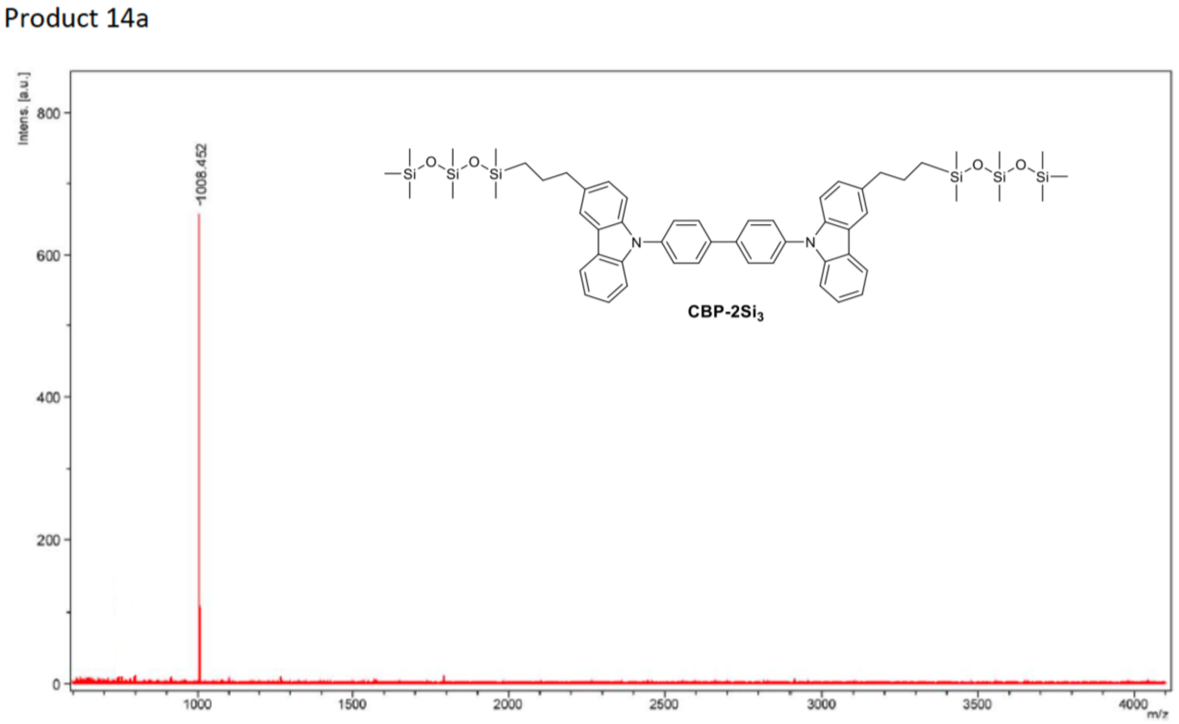

^{1}H NMR of (14b)

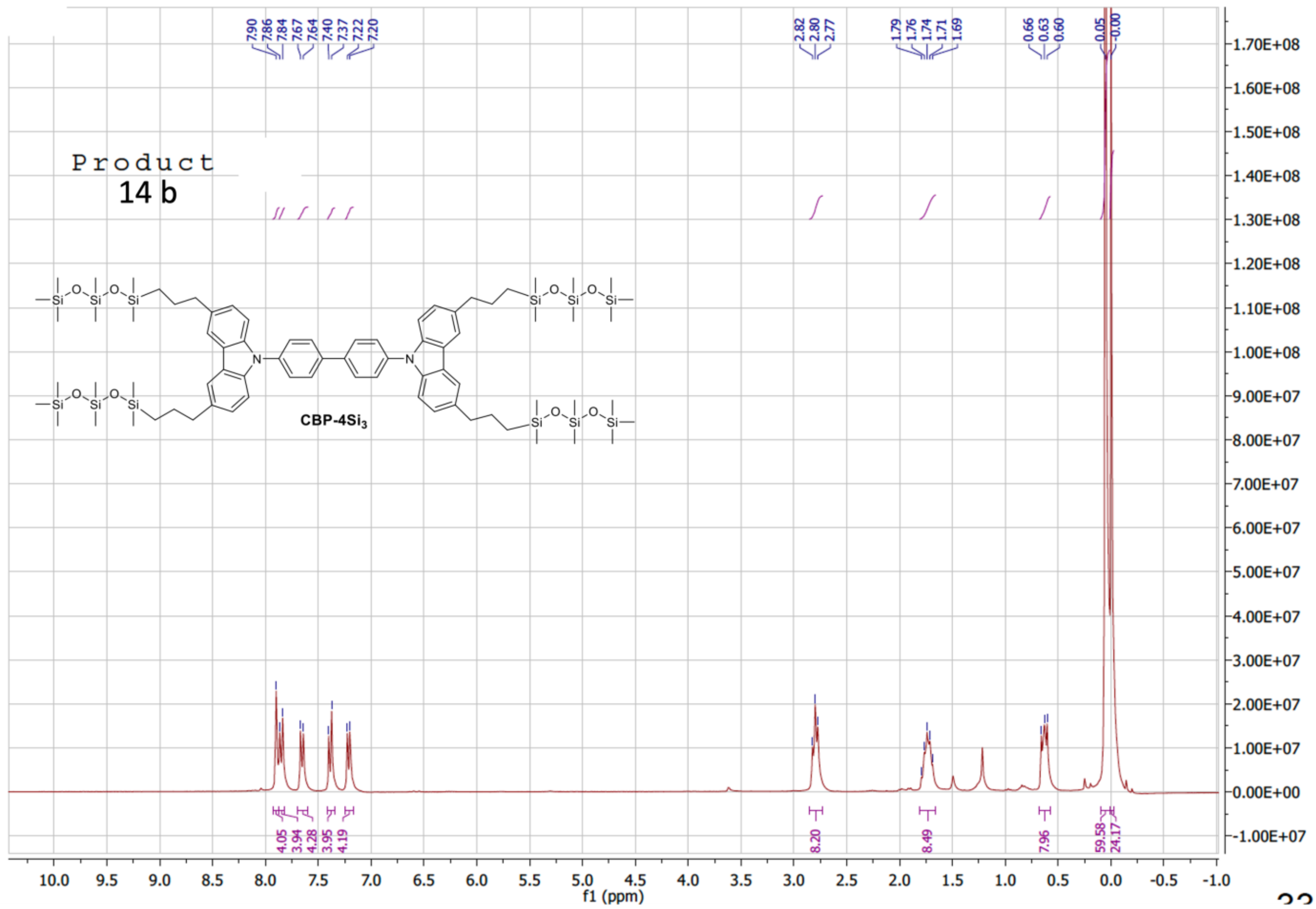

^{13}C NMR of (14b)

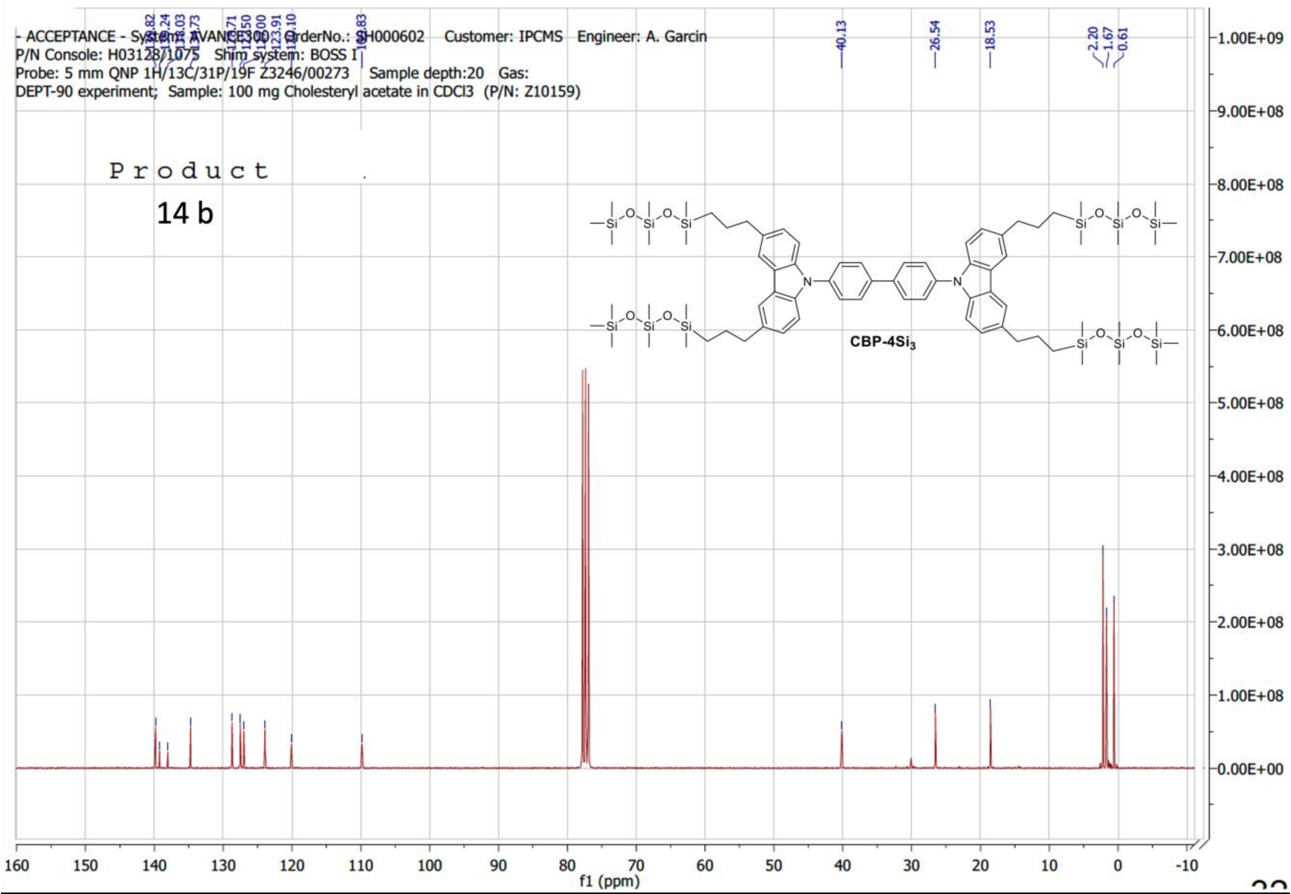

MALDI of (14b)

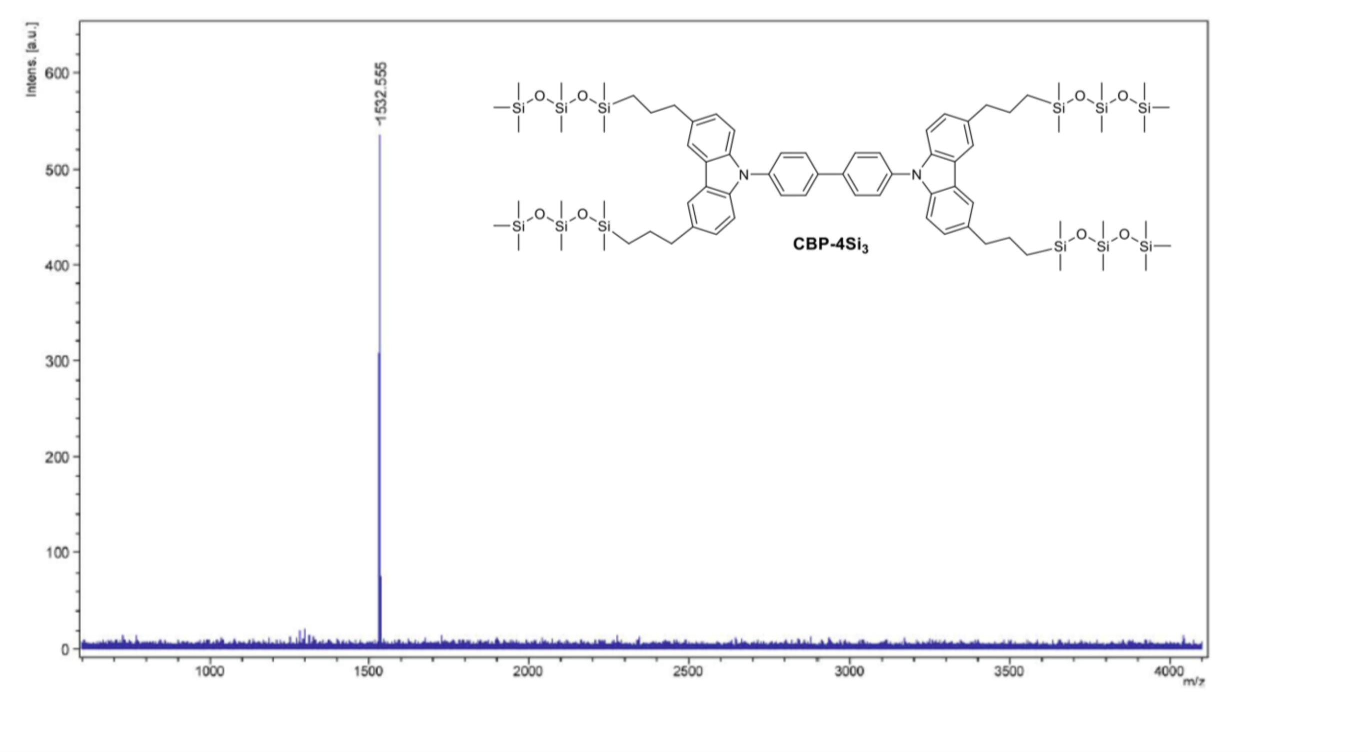
Intens. [a.u.]
1532.555
CBP-4Si$_3$
m/z
0
100
200
300
400
500
600
1000
1500
2000
2500
3000
3500
4000
